# TFEB activates Nrf2 by repressing its E3 ubiquitin ligase DCAF11 and promoting phosphorylation of p62

**DOI:** 10.1038/s41598-019-50877-8

**Published:** 2019-10-04

**Authors:** Jee-Yun Park, Sunhyo Kim, Hee Young Sohn, Young Ho Koh, Chulman Jo

**Affiliations:** Division of Brain Diseases, Center for Biomedical Science, Korea National Institute of Health, 187 Osongsaengmyeong 2-ro, Osong-eup, Cheongju-si, Chungcheongbuk-do 28159 Korea

**Keywords:** Stress signalling, Post-translational modifications

## Abstract

Transcriptional factor EB (TFEB) and nuclear factor E2-related factor 2 (Nrf2) play crucial roles in the biological response against cellular stressors; however, their relationship has not yet been investigated. Here, we constructed human neuroglioma cell lines stably expressing TFEB. The expression of Nrf2-response genes, including heme oxygenase (HO)-1, glutathione-s-transferase-*mu*1 (GSTM1), and p62, was induced in the cell line, independent of oxidative stress. Of note, the protein level of Nrf2 was significantly increased, and its ubiquitinated fraction was reduced in stable cells compared to that in the control cells. Among E3 ubiquitin ligases known to be involved in the ubiquitination of Nrf2, DDB1 and Cullin4 associated factor 11 (DCAF11) was down-regulated at both protein and mRNA levels in stable cells, indicating that the repression of DCAF11 by TFEB may be mainly involved in the stabilization of Nrf2. In addition, the level of phosphorylated p62 at S349 was highly increased in stable cells compared to that in control cells, which could allow it to interfere with the association of Keap1 and Nrf2, thus stabilizing Nrf2. We suggest for the first time that TFEB could activate Nrf2 by increasing its stability under conditions devoid of oxidative stress.

## Introduction

The transcriptional factor nuclear factor E2-related factor 2 (Nrf2) regulates genes involved in the cellular response against various stressors, including oxidative stress^1,2^. The activity of Nrf2 is tightly controlled by a complex array of transcriptional regulators and post-translational modifications to ensure its proper activity, both under basal conditions and during adaptation to environmental changes^3,4^. Nrf2 is a short-lived protein present in the cytoplasm under homeostatic conditions, and its protein level is primarily regulated by the ubiquitin-proteasome system^5,6^. The well-characterized mechanism for controlling Nrf2 activity at the protein level involves Keap1 (Kelch-like ECH-associated protein 1), which is known as an adaptor protein of Cul3/Rbx1 E3 ubiquitin ligase for Nrf2^7^. Under homeostatic conditions, two molecules of Keap1 bind to the ETGE and DLG motifs in the Neh2 domain of Nrf2 and recruit the Cul3/Rbx1complex, leading to its ubiquitination and subsequent proteasomal degradation^8–10^. Under oxidative stress, however, the cysteine residues (Cys151 or Cys273/288) on Keap1 react with electrophilic compounds, resulting in sulfhydryl bonding or adduct formation, which weakens its interaction with Nrf2 and thus rescues Nrf2 from the degradation^11–14^. Nrf2 moves into the nucleus, where it binds to antioxidant response element (ARE) sequences, thus activating genes encoding antioxidant, detoxification, anti-inflammation, proteasome, and autophagy proteins^2,15^.

In the last decade, the understanding of the molecular mechanism regulating Nrf2 stability has greatly advanced. In addition to Keap1, three kinds of E3 ubiquitin ligases have been found to participate in the degradation of Nrf2 independent of Keap1: β-transducin repeat-containing protein (β-TrCP), DDB1 and Cullin4 associated factor 11 (DCAF11, also referred to as WDR23), and HMG-CoA reductase degradation 1 homolog (Hrd1)^16–20^. β-TrCP interacts with the Neh6 domain phosphorylated by GSK-3β in Nrf2 together with the Skp1-Cul1/Rbx1 complex and leads to degradation of Nrf2 by the ubiquitin-proteasome system^16,17,20^. DCAF11 and DDB1-Cul4/Roc1 bind to the DIDLID sequence of the Neh2 domain of Nrf2 to mediate its degradation^19^. Recently, Hrd1, a multipass ER membrane protein also called synoviolin, was found in liver tissues of cirrhosis patients. Hrd1 is up-regulated in response to ER stress and mediates the degradation of Nrf2^18^.

The transcriptional factor EB (TFEB), a basic helix-loop-helix leucine zipper transcription factor, is a master regulator of the autophagy-lysosome pathway^21,22^. In normal conditions, TFEB is localized in the cytoplasm, which is mediated by its phosphorylation by mammalian target of rapamycin complex 1 (mTORC1) and ERK2^21,23,24^. In addition to phosphorylation, members of the 14-3-3 protein family bind to phosphorylated TFEB to sequester it in the cytoplasm^25^. In cellular conditions that are known to inactivate mTORC1 such as stress, starvation, and lysosomal inhibition, the phosphorylation of TFEB is inhibited, thus allowing it to move into the nucleus. Nuclear TFEB binds to the coordinated lysosomal expression and regulation (CLEAR) element of genes, thus inducing the expression of genes involved in processes such as autophagy, lysosomal biogenesis and lysosomal exocytosis^22,23^.

Growing evidence suggests that Nrf2 and TFEB would cooperate against cellular stresses and for protein homeostasis^15,26–28^. Both transcriptional factors were known to cooperatively participate in the clearance of phosphorylated tau, which is a component of neurofibrillary tangles in the patient brains of Alzheimer’s disease^29,30^. In a recent study, the activation of Nrf2 could induce numerous genes working in the autophagy-lysosome pathway, just as TFEB activates^15,31^. Interestingly, we previously observed that fisetin, a small flavonoid molecule, could activate Nrf2 as well as TFEB in cortical cells and primary cultured neurons^26^. The activation of TFEB was explained by fisetin-mediated inhibition of mTORC1^32,33^; however, the mechanism of how Nrf2 is activated remains to be investigated. Thus, it could be speculated that Nrf2 and TFEB share a relationship. To investigate this potential relationship, we constructed a human neuroglioma cell line stably expressing TFEB and examined whether Nrf2 activity is changed in these cells. Importantly, we found that TFEB highly enhances Nrf2 activity through its stabilization, which stems from the repression of Nrf2-specific E3 ubiquitin ligase, DCAF11 and the increase of p62/SQSTM1 (hereafter referred to p62) phosphorylated at S349. Here, we describe a novel molecular mechanism in which TFEB activates Nrf2 under conditions devoid of oxidative stress.

## Results

### TFEB activates Nrf2

**Figure 1:**
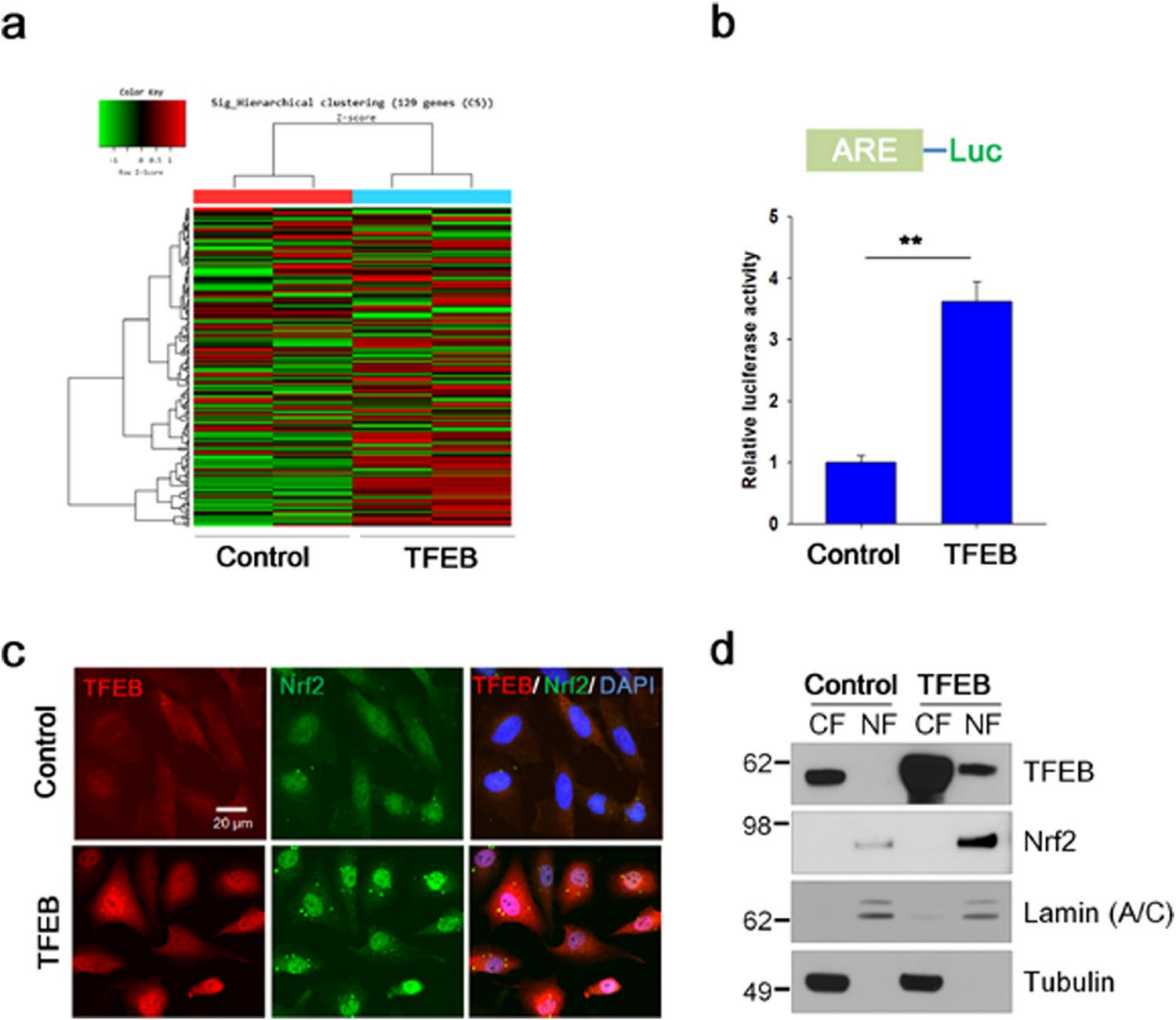
TFEB increases the transcriptional activity and the nuclear localization of Nrf2. (**a**) The gene expression levels in H4 cells (Control) and H4 cells stably expressing TFEB (TFEB) were analyzed using cDNA microarray as described in Methods. Heat map shows the expression levels for Nrf2-response genes. Shades of red represent up-regulation, and shades of green represent down-regulation. (**b**) H4 cells (Control) and H4 cells stably expressing TFEB (TFEB) were transiently transfected with the ARE-Luc reporter plasmid and assayed for the luciferase activity. (**c**) H4 cells stably expressing TFEB fixed with 4% paraformaldehyde were immunostained using anti-TFEB and Nrf2 antibodies. Fluorescence signals were observed using a fluorescence microscope. (**d**) Nuclear (NF) and cytosolic (CF) fractions from control and TFEB cells were prepared as described in Methods. The cellular locations of TFEB and Nrf2 were analyzed by immunoblotting using anti-TFEB and Nrf2 antibodies, respectively. To examine the purity of the fractionations, the blot was probed with antibodies to lamin (A/C), a marker for nuclear fraction, and tubulin, a marker for cytosolic fraction. Full blots are provided in Supplementary Fig. S10. Data shown are mean ± S.E. of four independent experiments and were analyzed using Student’s *t* test. (**p < 0.01).

**Figure 2:**
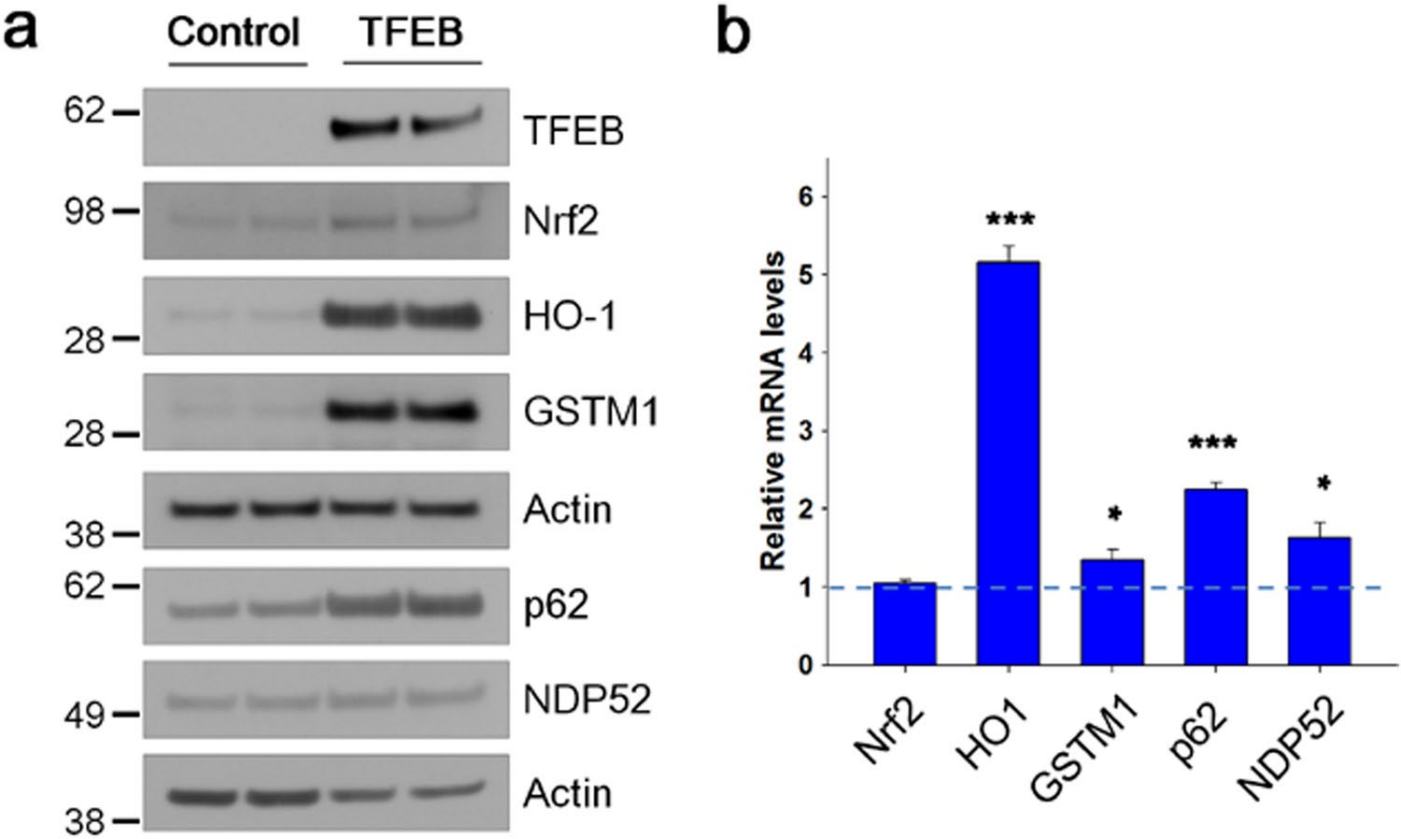
TFEB increases the expression of Nrf2-response genes. (**a**) The protein levels of Nrf2-response genes in H4 cells (Control) and H4 cells stably expressing TFEB (TFEB) were analyzed by immunoblotting using anti-TFEB, Nrf2, HO-1, GSTM1, p62, and NDP52 antibodies, respectively. Full blots are provided in Supplementary Fig. S10. (**b**) The mRNA levels of Nrf2-response genes were analyzed by qRT-PCR as described in Methods. Bar graph represents the relative mRNA level of genes in TFEB cells compared to H4 cells. Data shown are mean ± S.E. of three independent experiments and were analyzed using Student’s *t* test. (*p < 0.05; ***p < 0.001).

To examine whether TFEB could affect the transcriptional activity of Nrf2, we constructed human neuroglioma cells (H4) stably expressing TFEB (TFEB). First, we examined whether TFEB cells have a higher expression level in autophagic and lysosomal proteins. As shown in Supplementary Fig. S1a, a significant increase in mRNA levels of ATP6V1H, a component of vacuolar ATPase (V-ATPase), ATG9b, a regulator of autophagy, and lysosomal proteins such as lysosomal-associated membrane protein (LAMP) 1 and cathepsin D was seen in TFEB cells compared to H4 cells (Control). An increased protein level in lysosomal proteins was also observed in TFEB cells (Supplementary Fig. S1b), indicating that TFEB activity was highly induced in the cell line stably expressing TFEB. Then, the expression profile of genes in TFEB cells was examined by cDNA microarray analysis as described in Methods. A previous study suggested a set of genes induced in lymphoid cells by sulforaphane (SFN), a strong Nrf2 activator^34^. Among the genes with SFN-treated chromosome immunoprecipitation (ChIP)-seq peaks, we selected 121 genes not only with high confidence peaks (with more than 15 unique reads as described in the study) and at least one ARE element, but also induced above 1.0 fold (>1.0) by SFN (Supplementary Data gene set 1). To characterize the genes, we used the Database for Annotation, Visualization and Integrated Discovery (DAVID) functional annotation clustering tool^35,36^. Most of genes were annotated in functional catergories such as cellular oxidoreduction, and response to reactive oxygen species (ROS) (Supplementary Table S1). When the expression levels of the genes in TFEB cells were compared with those in H4 cells, 63.2% of genes was up-regulated in TFEB cells (Fig. 1a). The ARE-luciferase activity increased 3.6-fold in TFEB cells (Fig. 1b). Of note, the level of Nrf2 protein in TFEB cells increased, and it mainly localized in the nuclei (Fig. 1c,d), indicating that Nrf2 was activated. In addition, both the protein and mRNA levels of Nrf2 response genes, such as heme oxygenase (HO)-1, glutathione-s-transferase-*mu*1 (GSTM1), nuclear dot protein 52 (NDP52, also called as calcoco2), and p62, were also significantly augmented in TFEB cells (Fig. 2). Together, the results strongly suggest that TFEB could increase Nrf2 activity.

### TFEB does not induce ROS

**Figure 3:**
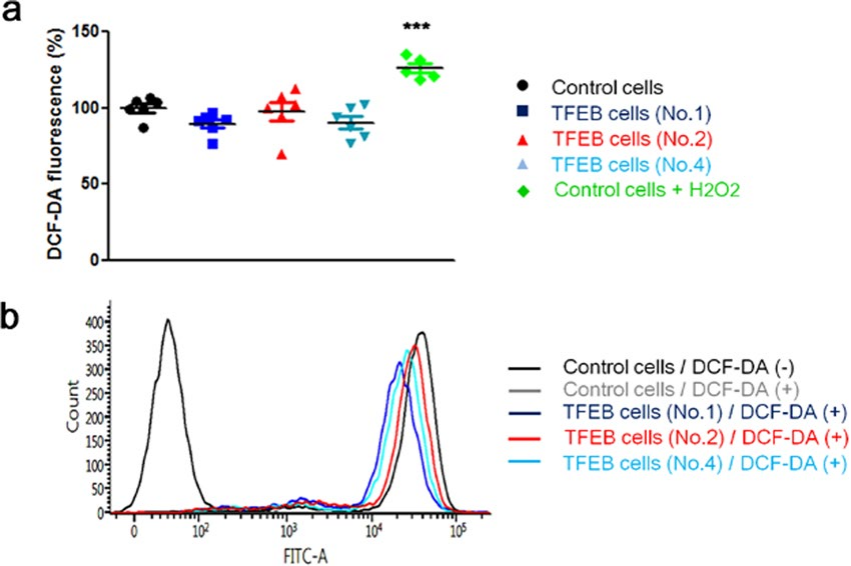
TFEB does not increase the cellular ROS level. The levels of intracellular ROS in H4 cells (Control cells) and H4 cells stably expressing TFEB (TFEB cells) were analyzed by measuring the fluorescence of DCF-DA. (**a**) Dot plot indicates the fluorescence intensities in the cells. As a positive control, 300 µM of hydrogen peroxide (H_2_O_2_) was treated to H4 cells for 1 h. (**b**) The cells were subjected to flow cytometry analysis as described in Methods for 10,000 cells. Data shown are mean ± S.E. of four independent experiments and were analyzed using Student’s *t* test. (***p < 0.001; *, cells treated with H_2_O_2_ versus cells not treated).

Nrf2 is activated in response to oxidative stress in cells. To confirm whether TFEB evokes a change in the intracellular level of ROS, we measured the fluorescence intensity of DCF-DA, which directly interacts with ROS, explaining the ROS level in cells. As shown in Fig. 3a, the fluorescence intensities in TFEB cells were not largely changed compared with those in the control cells, and significantly lower than those in cells treated with hydrogen peroxide (H_2_O_2_) as a positive control. Consistent with the result, the fluorescence intensities of TFEB cells in FACS analysis did not show a significant change compared to those in control cells (Fig. 3b). The result indicates that TFEB does not change intracellular ROS in cells. Thus, these results suggest that TFEB-mediated Nrf2 activation is not dependent on oxidative stress in cells.

### TFEB stabilizes Nrf2

**Figure 4:**
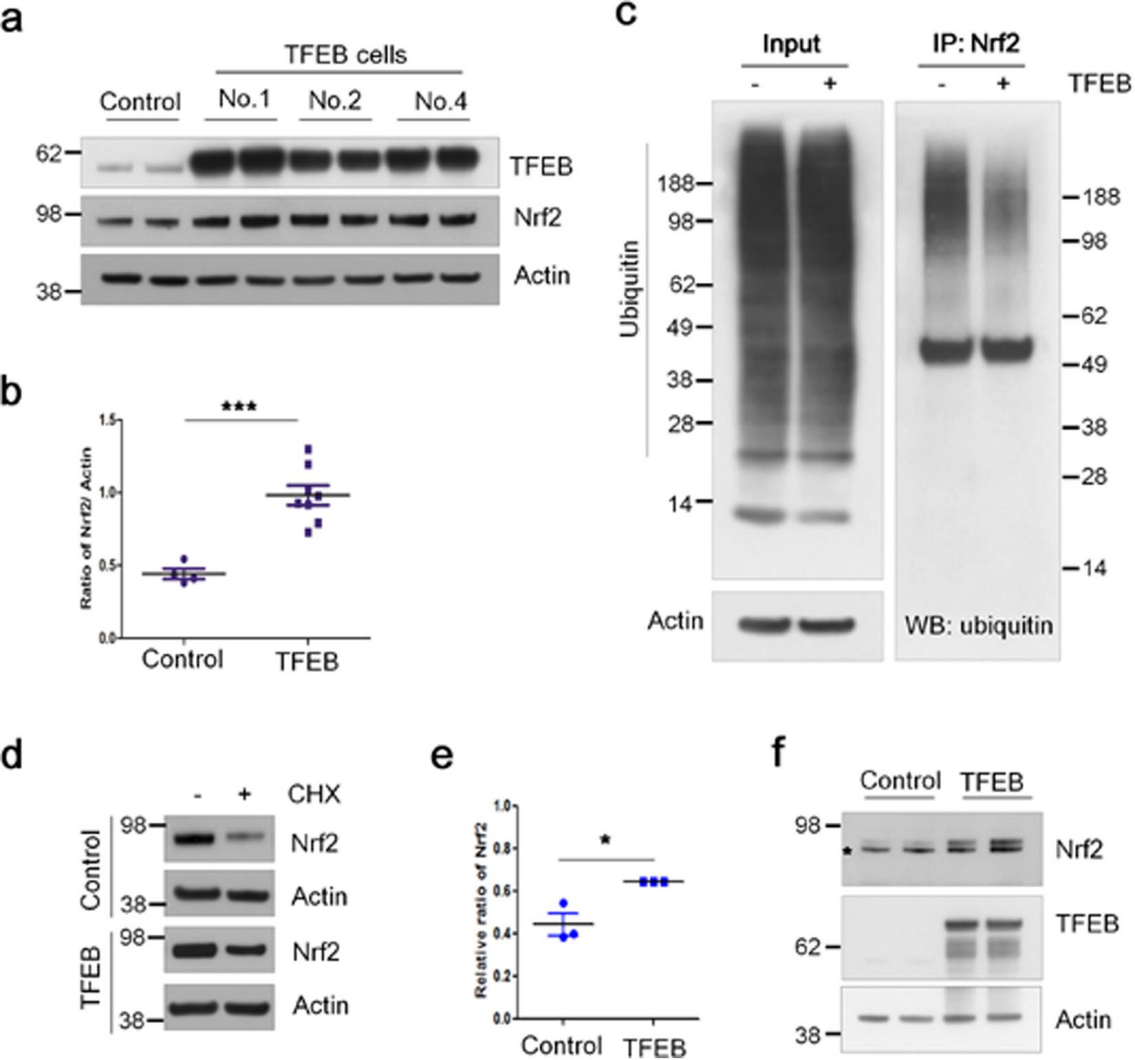
TFEB increases the stability of Nrf2. (**a**) The protein levels of TFEB and Nrf2 in H4 cells (Control) and H4 cell lines stably expressing TFEB (TFEB) were analyzed by immunoblotting using each corresponding antibody, respectively. (**b**) Dot plot represents the relative ratio of Nrf2 normalized with that of actin. (**c**) To examine the ubiquitination level of Nrf2 in H4 and TFEB cells, the cells were treated with 5 µM of MG132, a proteasome inhibitor, for 24 h. The cell lysates were used for the immunoprecipitation of Nrf2 using anti-Nrf2 antibody. The ubiquitination levels of Nrf2 were examined by immunoblotting using anti-ubiquitin antibody. (**d**) The cells were kept with or without 100 µM of cycloheximide (CHX) for 30 min. The protein levels of Nrf2 were analyzed by immunoblotting using anti-Nrf2 antibody. (**e**) Dot plot represents the relative ratio of Nrf2 in cells treated with CHX in contrast to those not treated. (**f**) HEK293 cells were transiently transfected with the Myc-Nrf2 expression plasmid and together with or without the pHM6-TFEB expression plasmid. The protein levels of Nrf2 and TFEB were analyzed by immunoblotting using anti-Myc and TFEB antibodies, respectively. The asterisk on the Nrf2 panel indicates a non-specific band. Full blots are provided in Supplementary Fig. S10. Data shown are mean ± S.E. of four independent experiments and were analyzed using Student’s *t* test. (*p < 0.05;  ***p < 0.001).

To examine how Nrf2 is activated in TFEB stable cells, we analyzed the protein levels of Nrf2. As shown in Fig. 4a,b, the levels of Nrf2 protein in TFEB cells increased about 2.2-fold compared with those in control cells. The increase in Nrf2 protein level was also observed in mouse embryonic fibroblast (MEF) cells transiently co-transfected with TFEB (Supplementary Fig. S2). As shown in Fig. 2b, the level of Nrf2 mRNA in TFEB cells was not changed compared with the control cells, indicating that TFEB does not affect its mRNA level. Nrf2 is primarily degraded via the ubiquitin-proteasome pathway, and its half-life is known to be approximately 20 min due to its high turnover^5,6^. To examine whether the stability of Nrf2 protein was changed in TFEB cells, we examined whether TFEB could change the ubiquitination level of Nrf2. Importantly, the ubiquitination level of Nrf2 was highly decreased in TFEB cells compared with that in control cells, demonstrating that TFEB could reduce the ubiquitination of Nrf2 (Fig. 4c). To further confirm whether the decreased ubiquitination of Nrf2 affects its half-life, the protein levels of Nrf2 were examined following the treatment of cycloheximide (100 μΜ), a protein synthesis inhibitor, to the cells for 30 min. The residual protein levels of Nrf2 in TFEB cells increased about 21% compared to control cells, indicating TFEB likely extends the half-life of Nrf2 through inhibiting its ubiquitination (Fig. 4d,e). In addition, we examined the levels of Nrf2 protein in HEK cells transiently co-transfected with plasmids expressing Nrf2 and TFEB. The protein levels of Nrf2 in cells co-transfected with TFEB increased without significant change in the mRNA levels (Supplementary Fig. S3), compared to those in control cells with the mock plasmid (Fig. 4f). Together, the results suggest that TFEB increases Nrf2 activity by enhancing its protein stability.

### TFEB represses DCAF11

**Figure 5:**
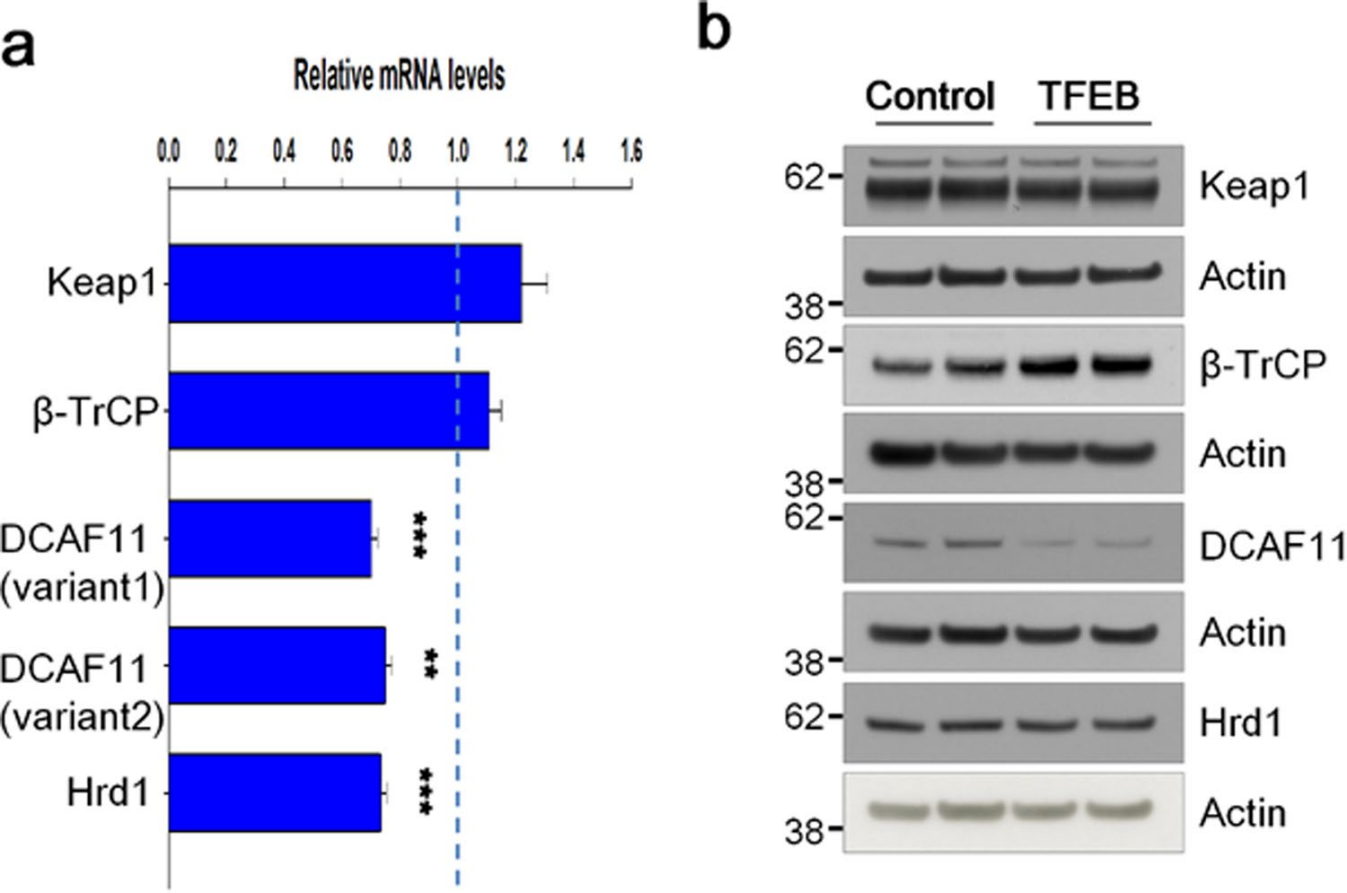
TFEB represses the expression of DCAF11. (**a**) The mRNA levels of Keap1, β-TrCP, DCAF11 and Hrd1 were analyzed by qRT-PCR as described in Methods. Bar graph represents the relative mRNA levels of Keap1 and Nrf2-specific E3 ubiquitin ligase genes in H4 cells stably expressing TFEB (TFEB) compared to H4 cells (Control). (**b**) The protein levels of Keap1 and Nrf2-specific E3 ubiquitin ligases in H4 and TFEB cells were analyzed by immunoblotting using each corresponding antibody. Full blots are provided in Supplementary Fig. S10. Data shown are mean ± S.E. of three independent experiments and were analyzed using Student’s *t* test. (**p < 0.01; ***p < 0.001).

**Figure 6:**
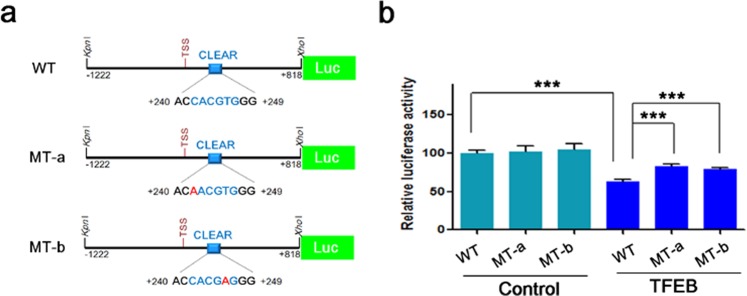
TFEB-mediated DCAF11 repression is dependent on the CLEAR element on its 5′-UTR. (**a**) shows a schematic drawing of luciferase-reporter plasmids with 5′-UTR of *DCAF11* gene having wild-type or mutant CLEAR element sequence. One CLEAR element is present at +242 ~ +247 from the transcription start site (TSS, +1). WT, wild-type; MT, mutant. (**b**) H4 cells (Control) and H4 cells stably expressing TFEB (TFEB) were transiently transfected with the indicated plasmid, and the luciferase activity was assayed at 18 h after transfection. Data shown are mean ± S.E. of four independent experiments and were analyzed using Student’s *t* test. (***p < 0.001).

The ubiquitination of Nrf2 is known to be regulated by Keap1, and three kinds of E3 ubiquitin ligases: β-TrCP, DCAF11, and Hrd1^16,18,19^. Since TFEB is a transcriptional factor, it is speculated that their transcriptional levels could be modulated by TFEB. Thus, we checked mRNA levels of Keap1, β-TrCP, DCAF11, and Hrd1 using quantitative real-time PCR as described in Methods. As shown in Fig. 5a, there was no significant change in mRNA levels of Keap1 and β-TrCP between control and TFEB cells; in contrast, however, mRNA levels of DCAF11 and Hrd1 decreased about 30% in TFEB cells compared with those in control cells. In accordance with this result, protein levels of DCAF11 were reduced in TFEB cells, whereas those of Hrd1 were not changed in the cells (Fig. 5b and Supplementary Fig. S4). To confirm whether DCAF11 is directly involved in the degradation of Nrf2, we examined the protein level of Nrf2 following the treatment of a *DCAF11*-specific siRNA into H4 cells. As shown in Supplementary Fig. S5, the protein level of Nrf2 was highly increased in the cells treated with a *DCAF11*-specific siRNA compared with that in cells with a scramble siRNA as control, indicating that DCAF11 is a functional E3-ubiquitin ligase for Nrf2. To further examine how DCAF11 is down-regulated in TFEB cells, we prepared luciferase-reporter plasmids in which 5′-untranslated region (UTR) of human *DCAF11* gene with wild-type or mutated CLEAR element sequence was cloned as described in Methods (Fig. 6a). As shown in Fig. 6b, the luciferase activity of the reporter plasmid with a wild-type CLEAR element sequence was dropped 36.9% in TFEB cells compared with that in H4 cells. Interestingly, the decrease in TFEB cells was significantly mitigated in case of plasmids with a mutant CLEAR element sequence, suggesting that TFEB likely inhibits the transcription of DCAF11 through the direct interaction of the CLEAR element on its 5′-UTR. These results demonstrate that the transcriptional repression of DCAF11 by TFEB might play a major role in the decreased ubiquitination of Nrf2, thus resulting in the increase of its stability.

### TFEB increases the phosphorylation of p62 at S349

**Figure 7:**
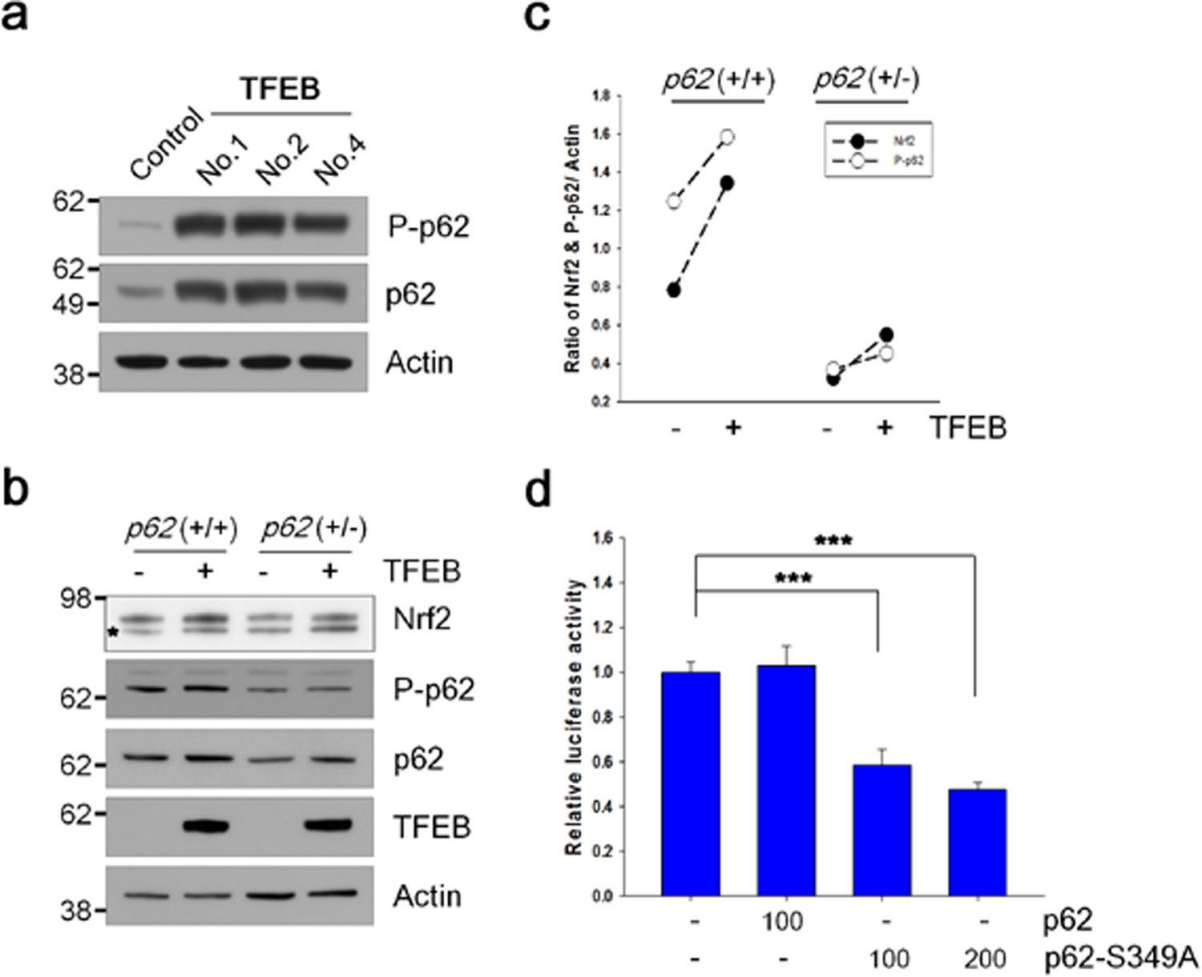
The phosphorylation of p62 at S349 is involved in TFEB-mediated Nrf2 activation. (**a**) The levels of phosphorylated p62 at S349 and total p62 in H4 cells (Control) and H4 cells stably expressing TFEB (TFEB) were analyzed by immunoblotting using anti-phospho-specific p62 (S349) and anti-p62 antibodies, respectively. (**b**) HEK 293 cells with the wild type or haplotype (+/−) *p62* gene were transiently transfected with the Myc-Nrf2 expression plasmid and together with or without the pHM6-TFEB expression plasmid. The protein levels of Nrf2, phosphorylated p62 at S349, p62 and TFEB were analyzed by immunoblotting using anti-Myc, phospho p62 (S349), p62 and HA antibodies. The asterisk on the Nrf2 panel indicates a non-specific band. (**c**) Scatter plot represents the relative ratio of Nrf2 and phosphorylated p62 at S349 normalized with that of actin. (**d**) H4 cells stably expressing TFEB were transiently transfected with the ARE-Luc reporter plasmid along with the wild-type or mutant p62 (S349A) plasmid. The number under graph indicates the amount of plasmids transfected in each well. The luciferase activity in the cells was measured following 24 h incubation. Full blots are provided in Supplementary Fig. S10. Data shown are mean ± S.E. of four independent experiments and were analyzed using Student’s *t* test. (***p < 0.001).

**Figure 8:**
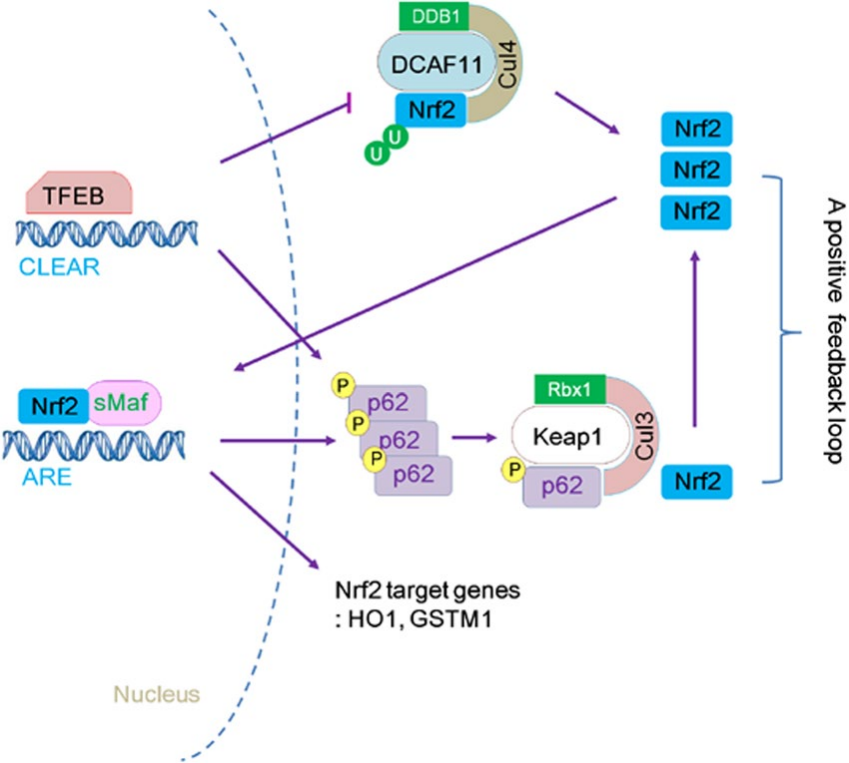
Schematic diagram showing the mechanism of Nrf2 activation by TFEB. TFEB first stabilizes Nrf2 through the repression of DCAF11, its E3 ubiquitin ligase, and activates Nrf2, resulting in the increased expression and phosphorylation of p62 at S349. Then, increased phosphorylated p62 disrupts the binding of Keap1 and Nrf2, thus leading to sustained Nrf2 activation through a positive feedback loop.

The protein level of p62 was highly increased in TFEB cells (Figs 2a and 7a). To clarify whether autophagy was impaired in TFEB cells, we checked the levels of LC3-II protein following the treatment of chloroquine, a lysosomal inhibitor. There was no significant difference in the levels of LC3-II accumulated by the inhibition of lysosomal degradation between TFEB and control cells (Supplementary Fig. S6), indicating that autophagy flux is intact in TFEB cells. The phosphorylation of p62 at S349 (S351 in mouse) could activate Nrf2 itself by sequestering Keap1 from Nrf2 due to its increased affinity for Keap1^37^. Thus, we examined the phosphorylation level of p62 at S349 using the phospho-specific p62 (S349) antibody. As shown in Fig. 7a, the phosphorylation levels of p62 at S349 were highly increased in TFEB cells compared with those in control cells. To examine how much the phosphorylation of p62 affects TFEB-mediated Nrf2 stabilization, we prepared a HEK 293 cell line with a haplotype *p62* gene (+/−) as described in Methods. The protein levels of Nrf2 protein induced by TFEB increased about 1.7-fold in both cells, irrespective of *p62* genotype (Fig. 7c). Intriguingly, the protein level of Nrf2 in cells with a haplotype *p62* gene (+/−) was lowered to 41% of wild type cells (Fig. 7b), explaining that TFEB-mediated Nrf2 stabilization is dependent on the level of phosphorylated p62 at S349. Next, we made use of the ARE-Luc and mutant p62 (S349A) plasmids. When the mutant p62 plasmid was co-transfected with the ARE-Luc plasmid in TFEB cells, the luciferase activity appeared to be significantly attenuated by the mutant but not by the wild-type p62 (Fig. 7d), demonstrating that the increased phosphorylation of p62 at S349 is involved in the activation of Nrf2 in TFEB cells. Thus, our results suggest that increased protein and phosphorylation levels of p62 induced by TFEB play a crucial role in TFEB-mediated Nrf2 activation (Fig. 8).

## Discussion

Nrf2 is tightly regulated at various levels, including transcription, translation, post-translation, and epigenetic modification, to cope with cellular stressors^3^. Here, we unveiled a novel role for TFEB activating Nrf2 through augmenting its stability. There was no difference in the level of Nrf2 mRNA (Fig. 2b), indicating that its transcriptional regulation is not involved in the novel activity of TFEB. Because the stabilization of Nrf2 was also observed by transient transfection of Nrf2 mammalian expression vector without an internal ribosomal entry site (IRES) at the 5′-UTR, which is known to be important in enhancing its translation together with TFEB^38^ (Fig. 4f), it appears that translational regulation is also not related to the increased level of Nrf2 protein. Thus, it is a type of post-translational regulation of Nrf2 because TFEB significantly decreases the ubiquitination level of Nrf2 by the repression of DCAF11 and the interference of Keap1 association with Nrf2 through the augment of phosphorylated p62 (Fig. 8).

p62 interacts with Keap1 via the DPSTGE amino acid sequence, which is similar to the ETGE motif on Nrf2^39^. The phosphorylation of p62 at S349 in the DP**S**TGE motif was known to be important for the interaction with Keap1^37^. As shown in Fig. 7a, the phosphorylation levels of p62 at S349 were significantly increased in TFEB cells compared to control H4 cells. The increased protein level of Nrf2 by TFEB transfection was highly dependent on the phosphorylation level of p62 (Fig. 7b,c). The induced activity of Nrf2 in TFEB cells was mitigated by transfection of the mutant p62 (S349A) plasmid (Fig. 7d). Thus, in addition to Nrf2 stabilization by TFEB-mediated repression of DCAF11, a positive feedback loop suggested by Jain *et al*.^40^ likely helps Nrf2 to be even further increased at its stability level, thus leading to the concomitant, sustained activation of Nrf2 (Fig. 8). Until now, several kinases including mTORC1, PKC-δ, and Tak1 (TGF-β activated kinase) have been known to phosphorylate p62 at S349^37,41,42^. PKC-δ seems to be not involved since its activity in TFEB cells was not largely different from that in control cells (Supplementary Fig. S7). It remains to be further examined what kind of kinase is mainly involved in the phosphorylation of p62 in TFEB cells.

Among Nrf2 specific E3-ubiquitin ligases, only the levels of β-TrCP protein were considerably increased in TFEB stable cell lines in contrast to control cells (Fig. 5). β-TrCP is known to recognize the phosphorylated DSGIS motif at Neh6 domain of Nrf2 by GSK-3β and tag it for proteasomal degradation by a Cul1/Rbx1 complex^17,20^. Thus, we examined whether the activation of GSK-3β was checked by means of measuring the phosphorylation level of its Ser-9 in the stable cells; however, there was no significant change compared with those in control cells (Supplementary Fig. S8). Thus, even though the level of β-TrCP protein increases by TFEB, it is not likely to affect the stability of Nrf2 in the cells. Here, TFEB repressed the expression of DCAF11 E3-ubiquitin ligase at both the mRNA and protein levels (Fig. 5). As shown in Fig. 6b, TFEB-mediated repression of DCAF11 seems to occur via its direct binding to the CLEAR element on its 5′-UTR. In addition, knockdown of DCAF11 using *DCAF11*-specific siRNA evoked a remarkable increase in Nrf2 protein level, explaining that DCAF11 is functional E3-ubiquitin ligase for Nrf2 (Supplementary Fig. S5). Thus, the decrease of DCAF11 likely plays a primary initiator in the activation of Nrf2 in TFEB cells. Then, activated Nrf2 enhances the expression of p62^40^, forming a positive feedback loop (Fig. 8). Until now, there was no report that TFEB could inhibit the expression of a gene at the transcriptional level; however, in HeLa cells that stably overexpress TFEB, seven down-regulated genes were detected by the microarray analysis of its transcriptome^22^. Our result also suggests a novel role of TFEB as a repressor in gene expression.

Both TFEB and Nrf2 were activated by treatment of fisetin, a small flavonoid molecule in cortical neuronal cells and rat primary cultured neurons^26^. TFEB was likely to be activated through the direct inhibition of mTORC1 by fisetin^26,32,33^; however, the mechanism of how Nrf2 is activated has yet to be clearly elucidated. When the level of intracellular ROS was measured in cells treated with fisetin, there was no significant change compared with that in untreated control cells (data not shown), indicating that fisetin does not work as a prooxidant like sulforaphane^43^. A study suggested in human umbilical endothelial cells that fisetin induces Nrf2 activity through the activation of PKC-δ and p38 signaling pathways^44^. Here, we could not exclude a possibility that two signaling pathways are involved in the stabilization of Nrf2 by TFEB. To examine it, we analyzed the phosphorylation levels of two kinases in TFEB cells; however, there was no significant difference in their phosphorylation levels between TFEB and control cells (Supplementary Fig. S7). Also, the ubiquitination of Nrf2 was remarkably lowered in TFEB cells (Fig. 4c), whereas it was not changed in cells treated with fisetin (data not shown). Thus, our results demonstrate that Nrf2 activation by fisetin may be elicited via a different kind of molecular mechanism with TFEB cells.

Genetic ablation of key molecules such as Beclin-1, ATG5, or ATG7 in the autophagy pathway results in the accumulation of p62^45–47^, which sequesters Keap1, thus activating Nrf2 (called as non-canonical p62-Nrf2 activation)^27,28,39^. Here, we also observed a dramatic increase of p62 in TFEB cells (Figs 2a and 7a); however, autophagy is likely to be intact in TFEB cells, because there was no significant difference in the accumulated LC3-II levels between TFEB and control cells (Supplementary Fig. S6). In addition, when the plasmid expressing tau protein, which is mainly known to be cleared via autophagy^29^, was transfected into the cells, the protein level of tau was considerably lower in TFEB cells than that in control cells (data not shown). The results support that autophagy is not only functional, but also activated in TFEB cells. The expression of p62 has been known to be highly induced by TFEB^21^ as well as Nrf2^40^. In line with the previous result, p62 mRNA was significantly induced in TFEB cells (Fig. 2b). Given that Nrf2 was highly activated in TFEB cells (Figs 1 and 2), it seems that a higher level of p62 protein in the cells is originated from the cooperative relationship between Nrf2 and TFEB (Fig. 8), but not autophagy dysfunction.

Increasing evidence suggests that Nrf2 protects cells against inflammation as well as oxidative stress^3^. Also, it has been known that Nrf2/HO-1 axis plays a crucial role in anti-inflammatory function^48^. As shown in Fig. 2, the expression of HO-1 was highly induced in TFEB cells compared to control cells, suggesting that inflammatory response might be changed in TFEB cells. So, we examined the protein and mRNA levels of cyclooxygenase-2 (COX-2), a key enzyme of inflammation mediator prostaglandin synthesis in inflamed tissues^49^, in TFEB cells. The expression of COX-2 was considerably repressed in TFEB cells in contrast to control cells (Supplementary Fig. S9). Given that the expression of COX-2 was reversely increased in Nrf2 knockout mice^50^, it is speculated that the decrease of COX-2 could be mediated by Nrf2 activated in TFEB cells. Thus, our results provide a novel, important clue that a therapeutic activation of TFEB could mitigate not only oxidative stress, but also inflammation.

Oxidative stress and misfolded proteins synergistically contribute to the pathogenesis of neurodegenerative diseases such as Alzheimer’s disease, Huntington’s disease and Parkinson’s disease^51^. Thus, our results suggest that TFEB is an effective, therapeutic target for neurodegenerative diseases due to its dual functions activating the Nrf2-ARE pathway as well as the autophagy pathway. A growing body of evidence, on the other hand, suggests that aberrant, constitutive activation of Nrf2 is correlated with tumorigenesis, chemoresistance, and radioresistance in cancer cells^52^. Considering it, the results give a warning that a therapeutic activation of TFEB should be carefully introduced according to diseases.

## Methods

### Antibodies, reagents, and plasmids

Anti-Nrf2 (12721), TFEB (4220), NDP52 (9036), β-TrCP (4394), Hrd1 (14773), HA (2367), and ubiquitin (3936) antibodies were purchased from Cell Signaling Technology. Anti-lamin (A/C) (SC-6215) and GSTM1 (SC-133641) antibodies were obtained from Santa Cruz Biotechnology. Anti-Nrf2 rat monoclonal antibody (14596) was purchased from Cell Signaling Technology. Anti-heme oxygenase (HO)-1 (ADI-SPA-895) and p62 (BML-PW9860) antibodies were purchased from Enzo Life Sciences. Anti-DCAF11 (NBP1-57691) antibody was obtained from Novus Biologicals. Anti-phospho p62 (S349, PM074) antibody was purchased from Medical & Biological Laboratories. Anti-tubulin (T6074) and β-actin (A5316) antibodies were obtained from Sigma and Millipore, respectively. Protease inhibitor cocktail (P8340) and other chemicals were purchased from Sigma. The plasmid expressing human TFEB was constructed by cloning the human TFEB gene, which was amplified from cDNA of HEK 293 cells, into *Kpn*I and *EcoR*I sites of the pHM6 plasmid (Roche). The luciferase-reporter plasmid with 5′-UTR (−1222 ~ +818) of *DCAF11* gene was prepared by cloning the PCR product amplified from human genomic DNA into *Kpn*I and *Xho*I sites of the pGL2-Basic plasmid (Promega). The human Myc-Nrf2 and ARE-Luc plasmids were described in previous studies^26,29^. The Myc-p62 plasmid was gifted from Dr. Terje Johansen. The Myc-p62-S349A plasmid and luciferase-reporter plasmids with a mutant CLEAR element sequence in 5′-UTR of *DCAF11* gene were prepared by using the QuickChange II Site-Directed Mutagenesis Kit (Agilent Technologies) according to the manufacturer’s protocol.

### Cell culture and stable cell line

Human neuroglioma (H4) and HEK 293 cells were cultured in Dulbecco’s modified Eagle’s medium (DMEM) supplemented with 10% fetal bovine serum (FBS), 10 units/ml penicillin, and 100 units/ml streptomycin at 37 °C in a humidified atmosphere containing 5% CO_2_. Mock cells and H4 cells stably expressing human TFEB were transfected with the pcDNA 3.1(+) and pHM6-TFEB plasmids, respectively, and established by G418 selection.

### cDNA microarray

Total RNA from cell lines was extracted using Trizol reagent (Invitrogen) according to the manufacturer’s protocol. Biotinylated cRNA (anti-sense RNA) was prepared using the TargetAmp-Nano Labeling Kit (Epicentre) according to the manufacturer’s instructions. Briefly, 500 ng of total RNA was reverse-transcribed to cDNA using a T7 oligo(dT) primer. Then, second-strand cDNA was synthesized, which was used for the synthesis of cRNA by *in vitro* transcription in the presence of biotin-NTP. After purification, the cRNA was quantified using the ND-1000 Spectrophotometer (NanoDrop). A total of 750 ng of labeled cRNA was hybridized to each Human HT-12 v4.0 Expression Beadchip (Illumina Inc.) for 18 h at 58 °C. Detection of array signal was carried out using Amersham fluorolink streptavidin-Cy3 (GE Healthcare Bio-Sciences) following the bead array manual. Arrays were scanned with an Illumina Bead Array Reader Microarray Scanner according to the manufacturer’s instructions.

### Transient transfection and luciferase assay

Cells were transiently transfected with the plasmids needed for each experiment using Lipofectamine 2000 (Invitrogen). The total amount of DNA used for each well was normalized with the relevant mock vectors. For the luciferase assay, H4 cells or TFEB cells stably expressing TFEB were transiently transfected with the luciferase-reporter plasmid together with the TK-renilla plasmid using Lipofectamine 2000 (Invitrogen). Luciferase activity was measured using the Luciferase assay system (Promega, E2920) and a Luminometer (GLOMAX, Promega). Transfection efficiency was normalized with renilla activity.

### Immunohistochemical staining

H4 cells stably expressing human TFEB were fixed with 4% paraformaldehyde for 20 min. The cells were permeabilized by incubation in 0.2% Triton-X-100 in PBS for 30 min and blocked in a PBS blocking solution (2% normal goat serum, 0.1% Triton-X-100 in PBS) for 1 h following a short washing with PBS. The cells were incubated with rabbit anti-TFEB [1:250] and rat anti-Nrf2 [1:250]) antibodies diluted in the blocking solution at 4 °C overnight. Then, the cells were incubated with donkey anti-rabbit Alexa594 and anti-rat Alexa488 conjugated antibodies (1:500) for 1 h at room temperature. The cover-slips were mounted on glass slides with ProLong® gold antifade reagent (Invitrogen, P36935) following three washes with PBS. Images were photographed using a fluorescence microscope (Carl Zeiss).

### Immunoblotting

Cells were washed once with PBS and lysed with modified RIPA buffer (10 mM Tris-HCl [pH 7.4], 150 mM NaCl, 1 mM EGTA, 1% NP-40, 0.25% sodium deoxycholate, 0.1% SDS) containing 1 mM NaF, 1 mM Na_3_VO_4_, and 1x protease inhibitor cocktail. Proteins were extracted on ice with periodic vortexing for 30 min, lysates were cleared by centrifugation at 10,000 × *g* for 10 min at 4 °C, and the supernatants were used for immunoblotting following boiling in 1x SDS-sample loading buffer and 1x reducing agent at 97 °C for 7 min. For analysis, protein samples (20–40 μg) were separated on NuPAGE^TM^ Bis-Tris Protein gels (Invitrogen) at a constant current of 20 mA followed by transfer to nitrocellulose membranes (GE Healthcare) and immunoblotting with the indicated antibodies. Blots were developed with chemiluminescence (Thermo scientific). All protein concentrations were determined using the BCA method (Sigma). The blots were analyzed using Image J software.

### Preparation of nuclear and cytosolic fractions

Cells  were washed with and scraped in PBS. Cell pellets were resuspended in fractionation buffer (10 mM HEPES [pH 7.9], 10 mM KCl, 1.5 mM MgCl_2_, 0.1% NP-40, 0.5 mM NaF, 200 mM Na_3_VO_4_, and 1x protease inhibitor cocktail). The cells were incubated on ice for 15 min with shaking. Lysates were centrifuged at 2,600 × g at 4 °C, and supernatants representing cytosolic fraction were collected. Subsequent to washing precipitates using the fractionation buffer without 0.1% NP-40, the precipitates then were resuspended with the modified RIPA buffer containing 1x protease inhibitor cocktail and incubated on ice for 20 min with periodic vortexing. The lysates were then cleared by centrifugation at 10,000 × g at 4 °C, and supernatants were used as the nucleic fractions.

### Quantitative real-time PCR

For the synthesis of cDNA, total RNA was isolated using RNAeasy® Mini Kit (Quiagen) according to the manufacturer’s protocol. cDNA was generated using 2 μg of RNA and RT-PCR EcoDry Premix (Clontech). Quantitative real-time PCR (qRT-PCR) was performed using the SYBR® Green Real-Time PCR Master Mix (Invitrogen, 4344463) on a Real-Time PCR Detection System (QuantStudio 6 Flex, Applied Biosystems). Each reaction consisted of 50 ng of cDNA product from the diluted reverse transcription reaction, 0.2 µM of primers (Supplementary Table S2), and 10 µl of SYBR® Green Real-Time PCR Master Mix. The reactions were incubated in a 96-well plate at 95 °C for 10 min, followed by 40 cycles of 95 °C for 15 s and 60 °C for 1 min. After the reactions were completed, the threshold was manually set and the threshold cycle (CT) was automatically recorded. The CT is defined as the fractional cycle number at which the fluorescence signal passes the fixed threshold. All reactions were run in three replicates for each sample.

### Immunoprecipitation

Cells were lysed in NP-40 lysis buffer (0.5% NP-40, 10 mM Tris-HCl [pH 8.0], 150 mM NaCl, 10 mM sodium pyrophosphate, 1 mM EDTA) containing 1 mM NaF, 1 mM Na_3_VO_4_, and 1x protease inhibitor cocktail. Equal amounts of lysates were incubated with 2 μg of antibodies pre-conjugated with sheep anti-mouse or rabbit magnetic beads (DYNAL, Invitrogen) for 3 h on a rotational shaker at 4 °C. After overnight incubation at 4 °C, the beads were washed with three changes of NETN wash buffer (0.1% NP-40, 50 mM Tris-HCl [pH 8.0], 150 mM NaCl, 1 mM EDTA) and boiled in 1x SDS-sample loading buffer and 1x reducing agent at 97 °C for 7 min. Proteins were separated by SDS-PAGE and immunoblotted as described above.

### ROS measurement

Cells were incubated with 10 μΜ of 6-carboxy-2′,7′-dichlorodihydrofluorescien diacetate (DCF-DA, Sigma) in culture media without serum for 30 min. After washing cells three times using PBS, the intracellular fluorescence was measured at excitation 495 nm and emission 529 nm using a microplate reader (SpectraMax 190, Molecular Devices). For the analysis of flow cytometry, the cells were washed twice using PBS, detached with trypsin, and resuspended in PBS. The cellular fluorescence was analyzed at the channel for FITC (BD FACSVerse Instrument).

### *p62* haplotype (+/−) cell

HEK 293 cells were transfected with the GeneArt® CRISPR Nuclease Vector (Life Technologies, A21175) with human *p62*-specific target sequence (5′-GTGAGCGACGCCATAGCGAG-3′). On the next day, cells with the plasmid were enriched using Dynabeads® CD4 magnetic beads. The cells with the haplotype (+/−) *p62* gene were screened by the PCR method^53^, and the cell was confirmed by sequencing.

## Supplementary information

Supplementary Information

Supplementary Data gene set 1
