# Supplementary material for: TFEB activates Nrf2 by repressing its E3 ubiquitin ligase DCAF11 and promoting phosphorylation of p62
Source: Sci Rep. 2019 Oct 4;9:14354. doi: 10.1038/s41598-019-50877-8 (PMC6778067; doi:10.1038/s41598-019-50877-8)
Supplement: Supplementary file 1 — Supplementary Information [file 41598_2019_50877_MOESM1_ESM.pdf]

TFEB activates Nrf2 by repressing its E3 ubiquitin ligase DCAF11 and promoting phosphorylation of p62

Jee-Yun Park, Sunhyo Kim, Hee Young Sohn, Young Ho Koh, and Chulman Jo

Supporting Information

Supplementary Methods

Cell culture. Mouse embryonic fibroblast (MEF) cells were cultured in Dulbecco's modified Eagle's medium (DMEM) supplemented with 10% fetal bovine serum (FBS), 10 units/ml penicillin, and 100 units/ml streptomycin at 37 °C in a humidified atmosphere containing 5% CO_2.

Antibodies. Anti-LAMP1 antibody was purchased from BD Biosciences. Anti-cathepsin D (2284), phospho-GSK-3β (5558), LC3B (3868), phospho-p38 (4511), p38 (9212), phospho-PKC-δ (9374), PKC-δ (9616), and COX-2 (4842) antibodies were purchased from Cell Signaling Technology.

Knockdown of *DCAF11* gene. For knockdown of *DCAF11* gene, a duplex *DCAF11* (5'-CUCACUUCUUG CCCAAUGATT-3') specific siRNA or scrambled RNA as a control was mixed with Lipofectamine® RNAiMAX (Invitrogen, 13778-150) in serum-free Opti-MEM medium for 10 min. The mixture was loaded onto H4 cells. After 48 h, the cells were lyzed as described in Methods .

Supplementary Figures

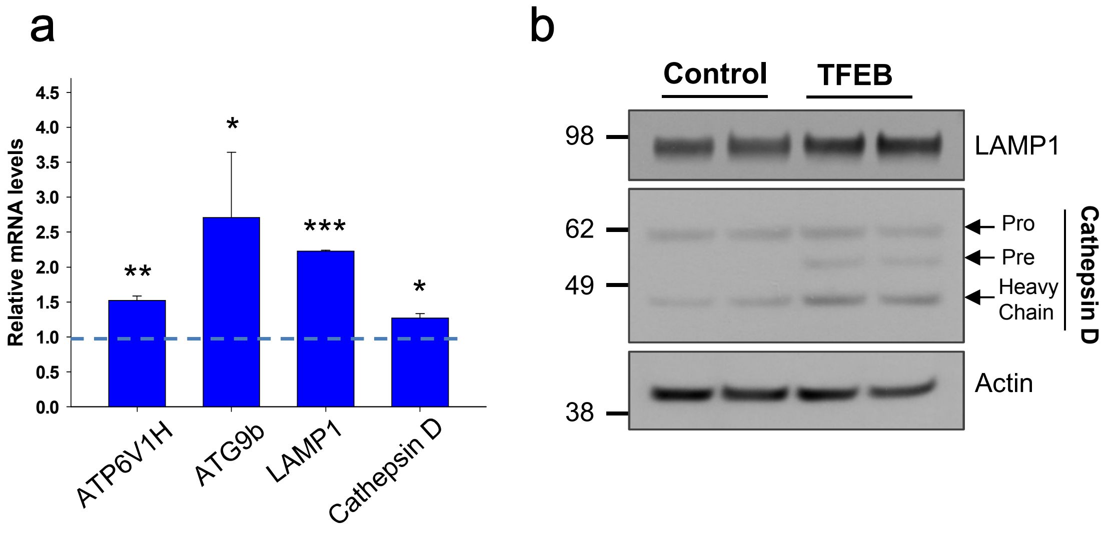

Supplementary Fig. S1. The expression levels of proteins in the autophagy-lysosome pathway increased in H4 cells stably expressing TFEB. (a) The mRNA level of proteins involved in the autophagy-lysosome pathway was analyzed by qRT-PCR as described in Methods. Bar graph represents the relative mRNA level in H4 cells stably expressing TFEB (TFEB) compared to H4 cells. (b) The protein levels of Lamp1 and cathepsin D in H4 (Control)and TFEB cells were analyzed by immunoblotting using anti-Lamp1 and cathepsin D antibodies, respectively. Full blots are in Fig. S10. Data shown are mean ± S.E. of three independent experiments and were analyzed using Student's *t* test. (*$p < 0.05$; **$p < 0.01$; ***$p < 0.001$)

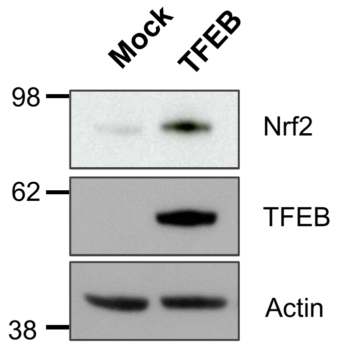

Supplementary Fig. S2. Ectopic expression of TFEB increases Nrf2. The plasmid expressing HA-tagged TFEB (TFEB) or pcDNA3.1(+) (Mock) was transiently transfected into mouse embryonic fibroblast (MEF) cells. The protein levels of Nrf2 and TFEB was analyzed by immunoblotting using anti-Nrf2 and HA antibodies, respectively. Full blots are in Fig. S10.

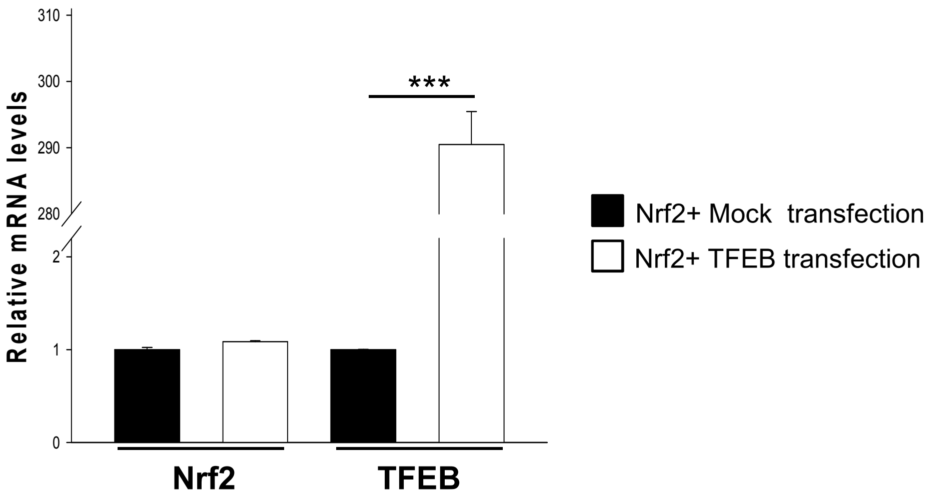

Supplementary Fig. S3. There was no significant change in Nrf2 mRNA level by co-transfection of TFEB . HEK 293 cells were transiently co-transfected with plasmids expressing Myc-Nrf2 and Mock plasmid (pcDNA3.1) or Myc-Nrf2 and TFEB. The mRNA level of Nrf2 and TFEB was analyzed by qRT-PCR as described in Methods. Bar graph represents the relative mRNA levels of Nrf2 and TFEB in HEK cells. Data shown are mean ± S.E. of three independent experiments and were analyzed using Student's *t* test. (***$p < 0.001$)

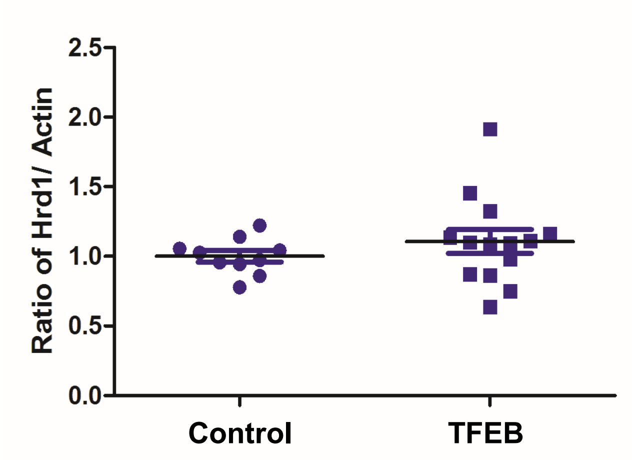

Supplementary Fig. S4. **TFEB does not affect the protein level of Hrd1.** The protein levels of Hrd1 in H4 cells (Control) and H4 cells stably expressing TFEB (TFEB) were analyzed by immunoblotting using the anti-Hrd1 antibody. Dot plot represents the relative ratio of Hrd1 normalized with that of actin.

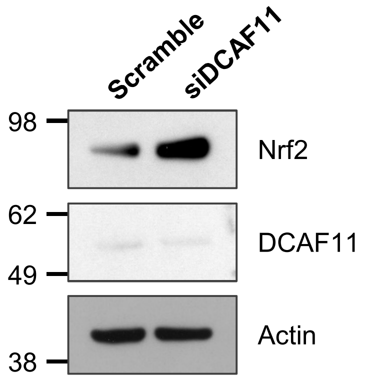

Supplementary Fig. S5. **Knockdown of *DACF11* gene increases the protein level of Nrf2.** An human *DCAF11*-specific siRNA was transiently transfected into H4 cells using RNAi MAX as described in Supplementary Methods. The protein levels of Nrf2 and DCAF11 were analyzed by immunoblotting using anti-Nrf2 and DCAF11 antibodies, respectively. Full blots are in Fig. S10.

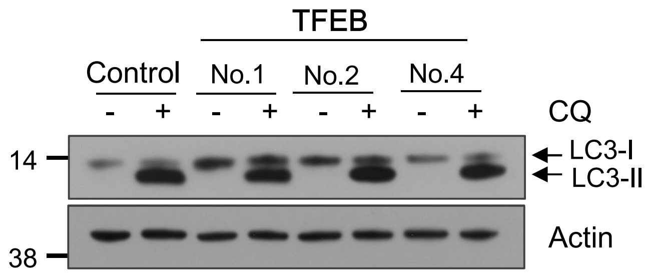

Supplementary Fig. S6. Autophagy is functional in TFEB cells. H4 cells (Control) and H4 cells stably expressing TFEB cell lines (TFEB) were cultured in the presence or absence of chloroquine (CQ, 50 μM) for 16 h. The protein levels of LC3B were analyzed by immunoblotting using each corresponding antibody. Full blots are in Fig. S10.

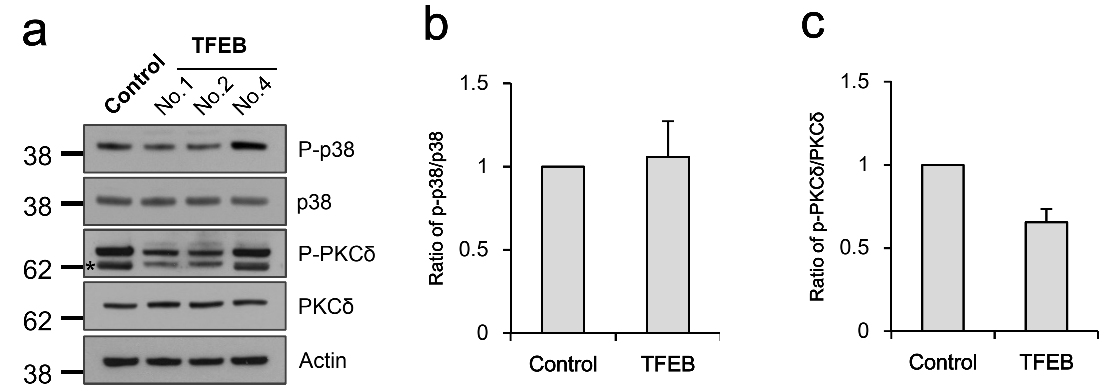

Supplementary Fig. S7. The p38 & PKC-δ signaling pathways are not involved in TFEB-mediated Nrf2 activation. (a) The phosphorylation levles of p38 & PKC-δ kinases in H4 cells (Control) and H4 cells stably expressing TFEB cell lines (TFEB) were analyzed by immunoblotting using each corresponding antibody. The asterisk on the P-PKC-δ panel indicates a non-specific band. Full blots are in Fig. S10. Bar graphs show the relative ratio of phosphorylated p38 (b) and PKC-δ (c) normalized with those of p38 and PKC-δ, respectively .

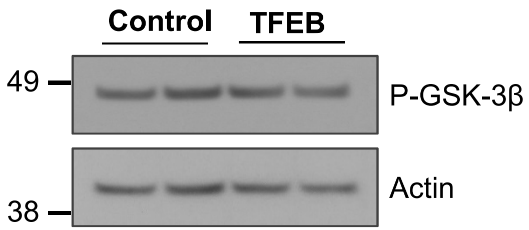

Supplementary Fig. S8. TFEB does not affect the phosphorylation level of GSK-3β. The phosphorylation level of GSK-3β at Ser-9 in H4 cells (Control) and H4 cells stably expressing TFEB (TFEB) were analyzed by immunoblotting using the phospho-specific GSK-3β antibody. Full blots are in Fig. S10.

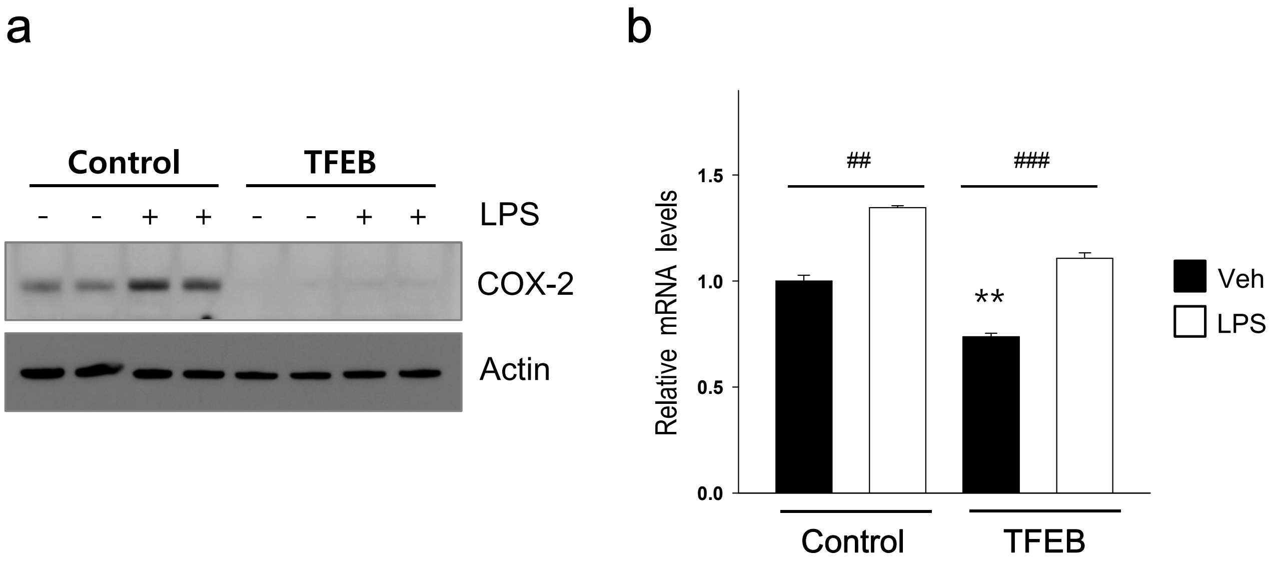

Supplementary Fig. S9. TFEB inhibits the expression of LPS-mediated COX-2. H4 cells (Control) and H4 cells stably expressing TFEB cell lines (TFEB) were treated with LPS (500 ng/ml) for 24 h. (a) The protein levels of COX-2 were analyzed by immunoblotting using anti-COX2 antibody. Full blots are in Fig. S10. (b) The mRNA level of COX-2 was examined by qRT-PCR described in Methods. Data shown are mean ± S.E. of three independent experiments and were analyzed using Student's *t* test. ($^{**}p < 0.01$; $^{\#\#}p < 0.01$; $^{\#\#\#}p < 0.001$; $^{\#}$, cells treated with LPS versus cells not treated)

Fig. 1d

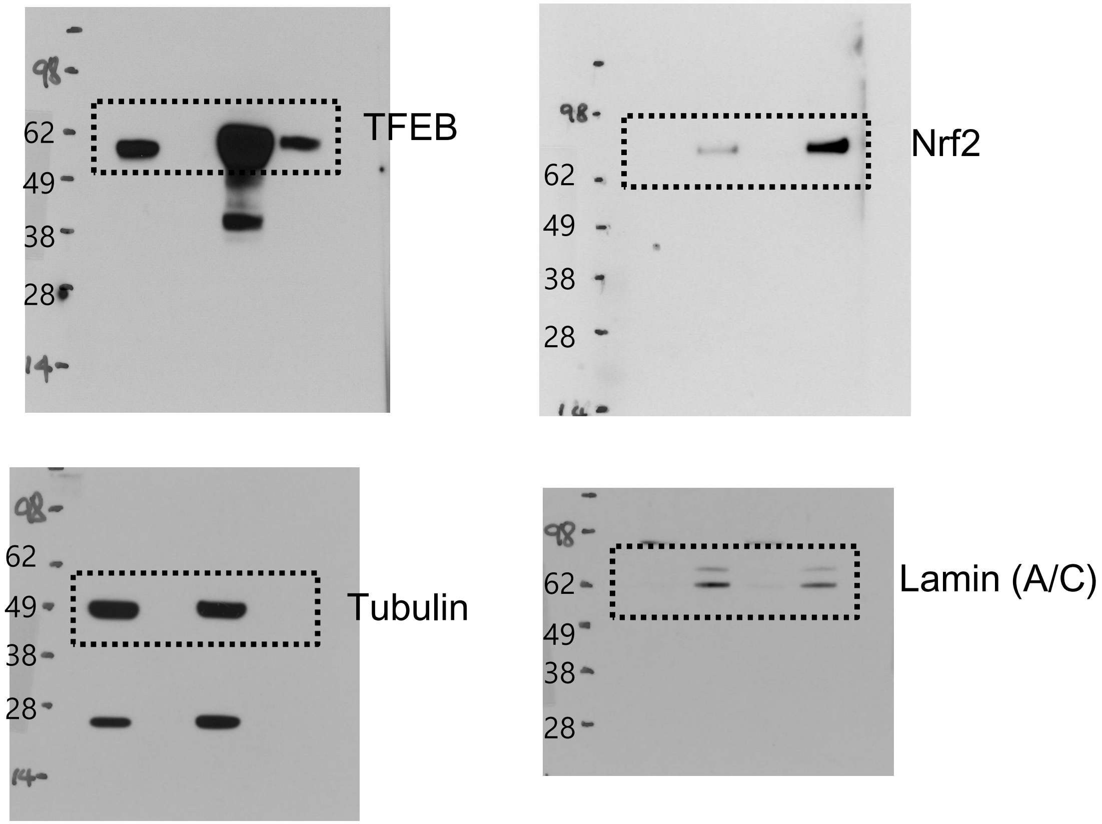

Supplementary Fig. S10. Full scans of uncropped blots .

Fig. 2a

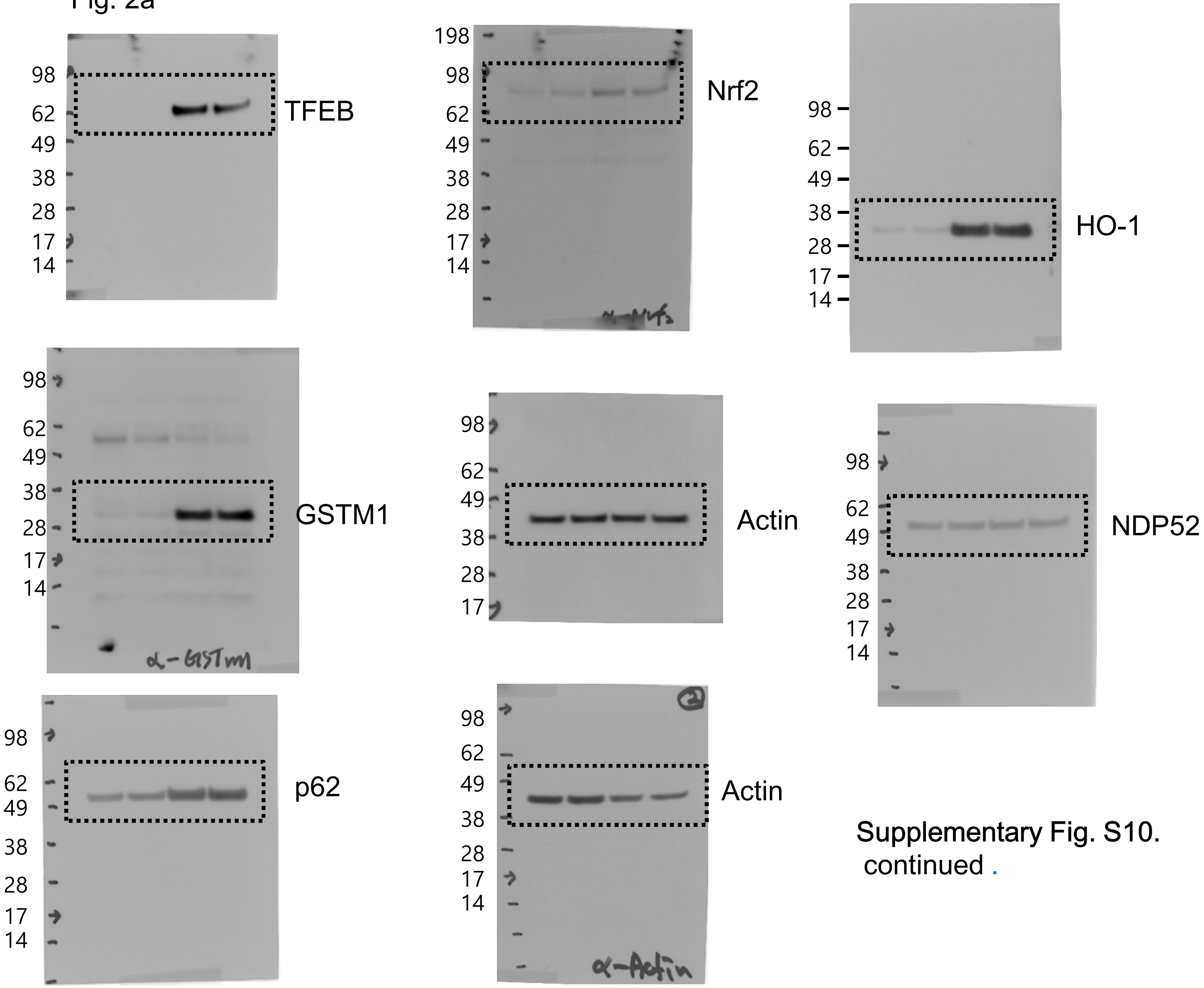

Supplementary Fig. S10.
continued .

Fig. 4a

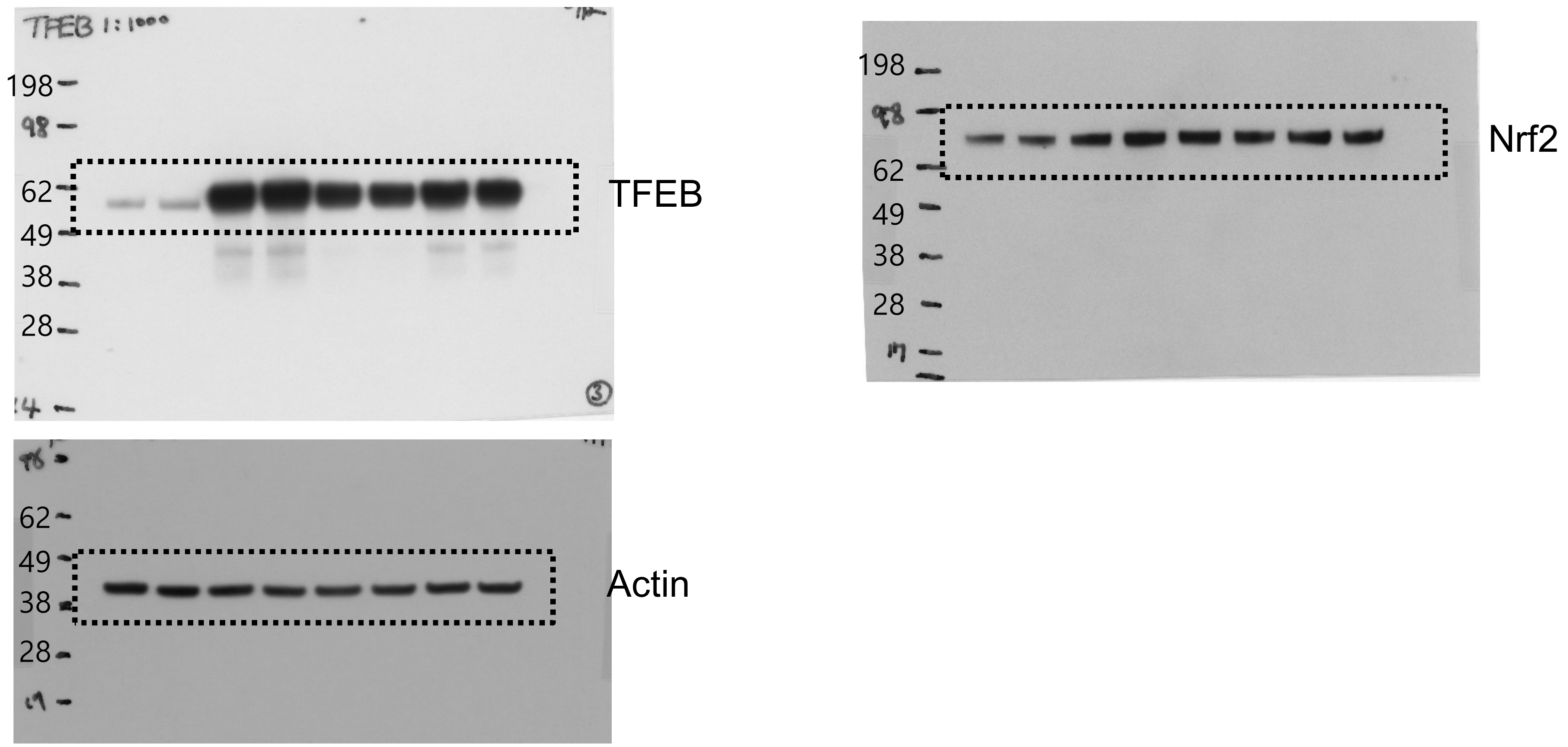

Fig. 4d

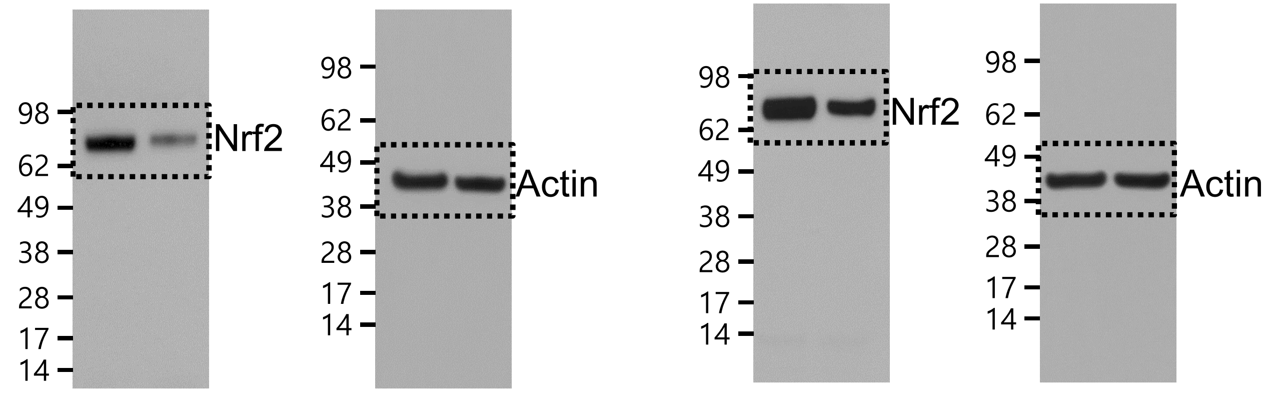

Supplementary Fig. S10. continued .

Fig. 4f

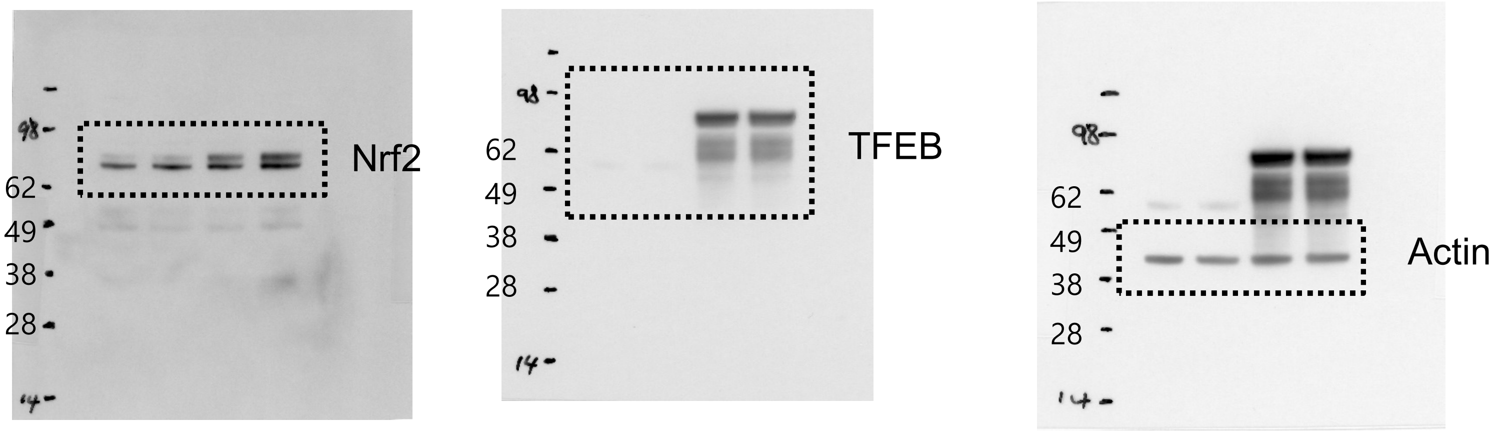

Supplementary Fig. S10. continued .

Fig. 5b

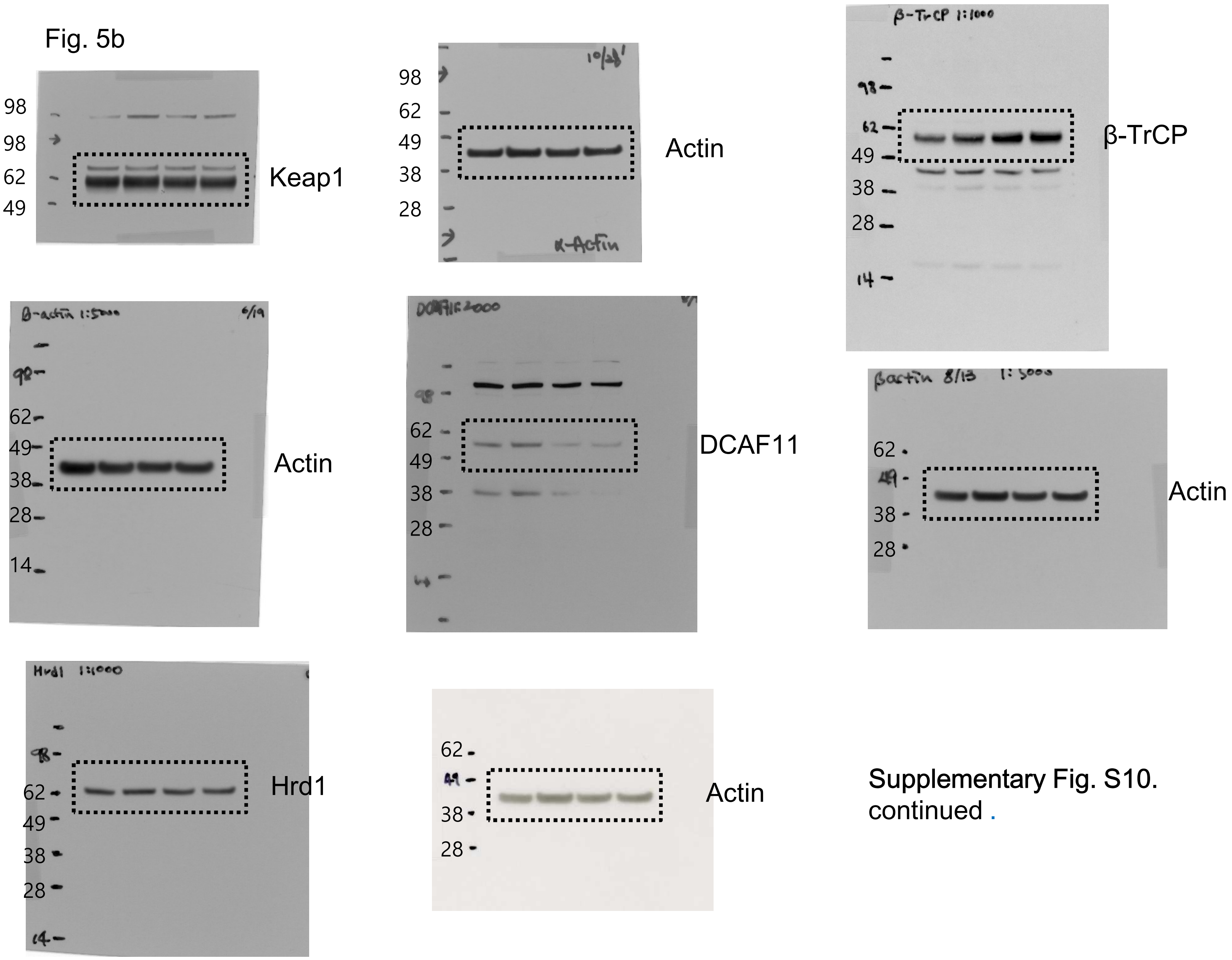

Supplementary Fig. S10. continued .

Fig. 6a

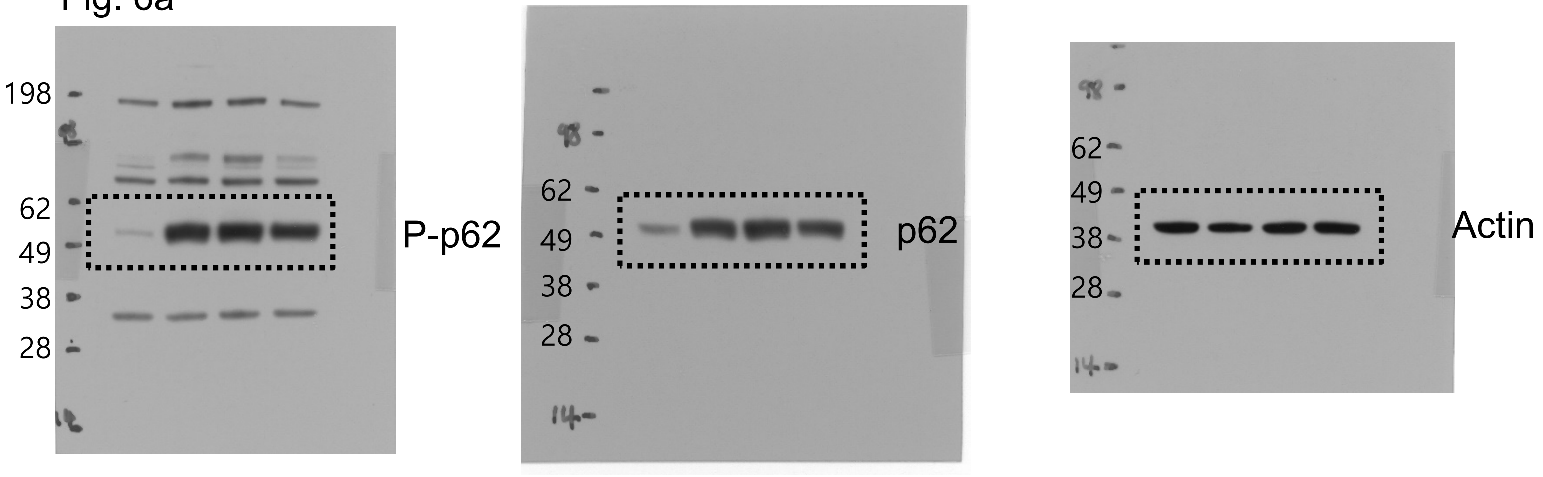

Fig. 6b

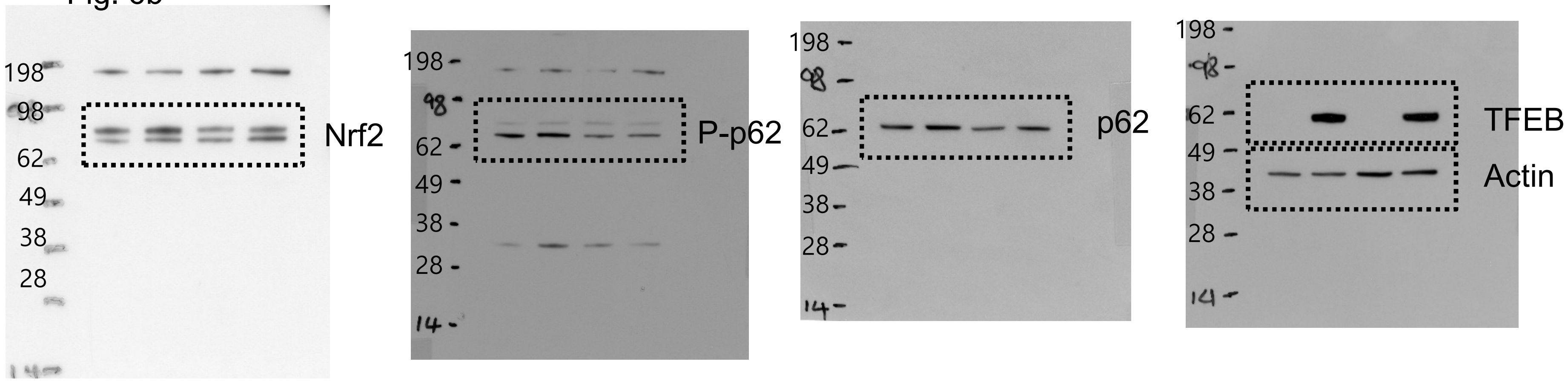

Supplementary Fig. S10. continued .

Fig. S1b

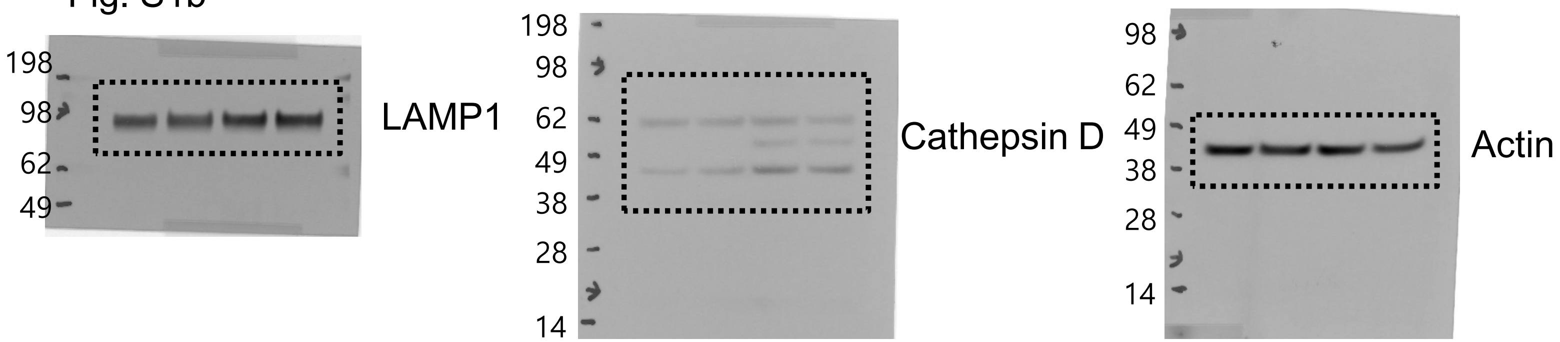

Fig. S2

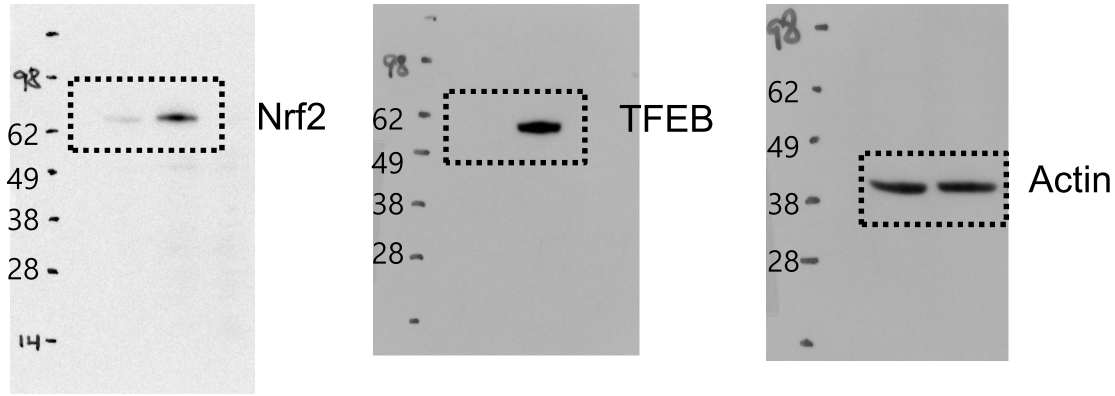

Supplementary Fig. S10. continued .

Fig. S5

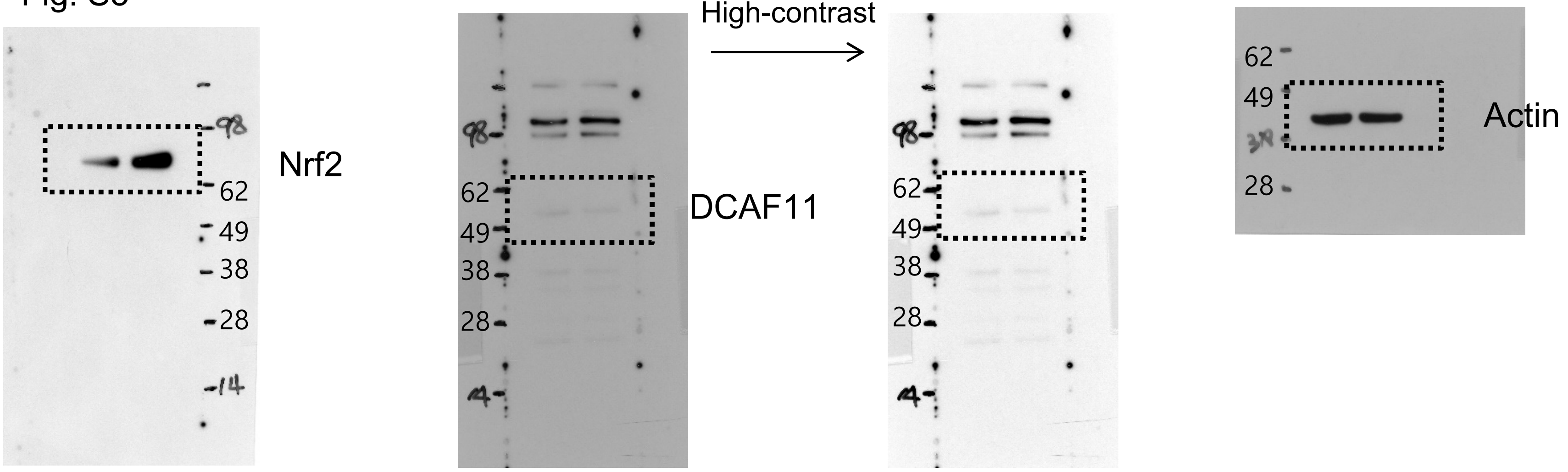

Fig. S6

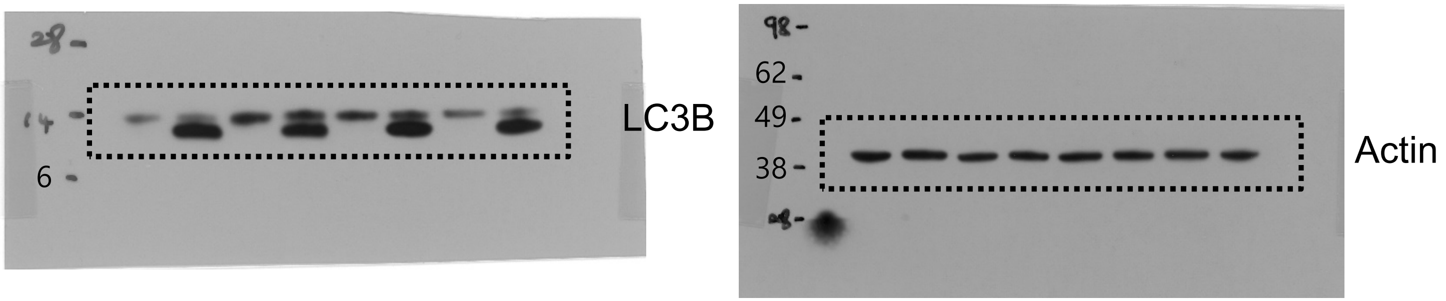

Supplementary Fig. S10. continued .

Fig. S7a

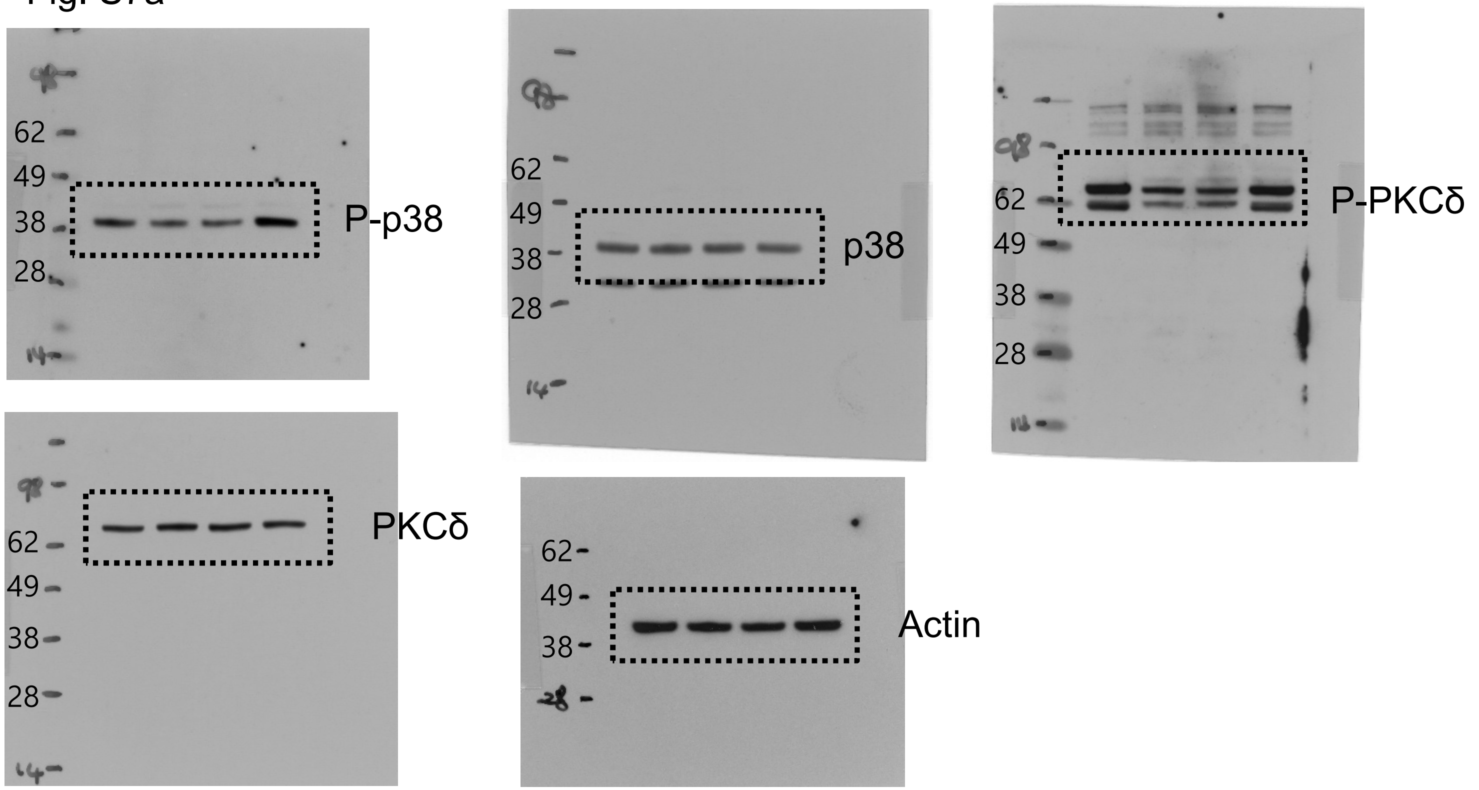

Fig. S8

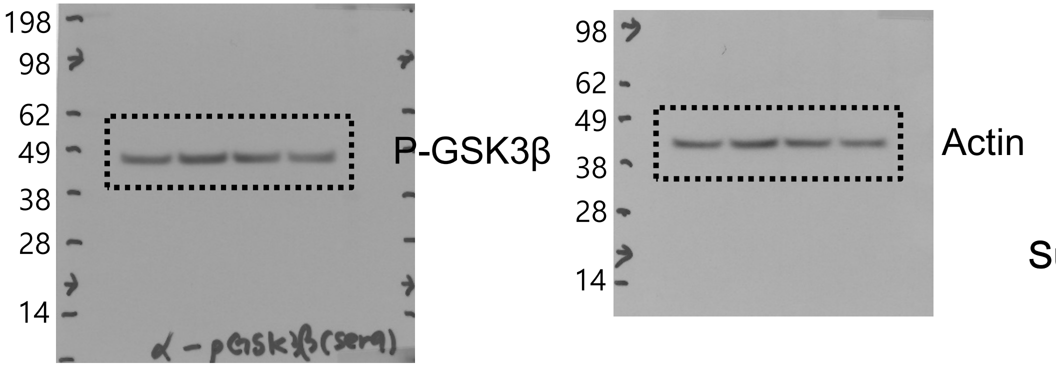

Supplementary Fig. S10. continued .

Fig. S9a

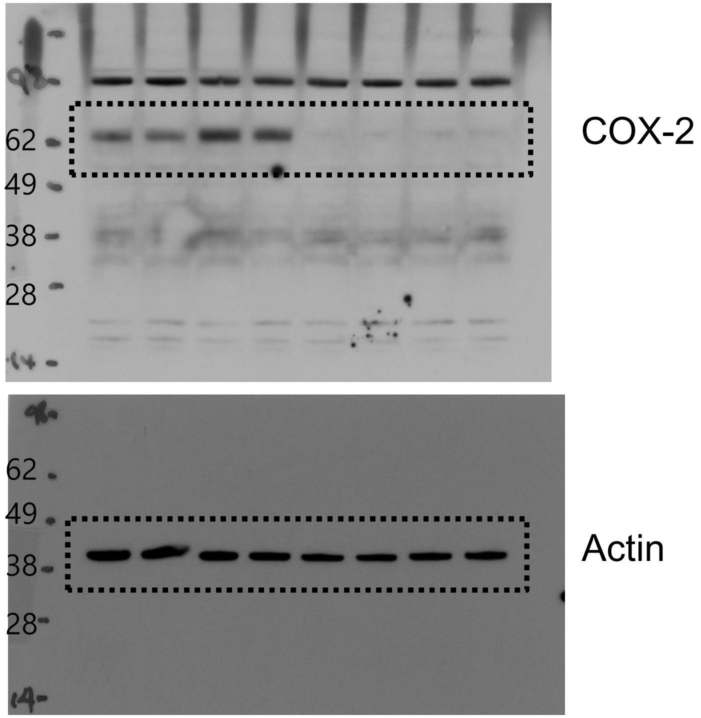

Supplementary Fig. S10. continued .

Supplementary Table

Supplementary Table S1. DAVID functional annotation clustering of 121 genes*

Clustered category	Enrichment score	Number of genes
Oxidoreductase	3.27	121
Response to reactive oxygen species	2.38	115
Protein processing in endoplasmic reticulum	2.14	71
RmlC-like cupin domain	1.80	116

* See Supplementary Data gene set 1 for gene lists and individual categories.

Supplementary Table S2. The sequence of primers used for qRT-PCR

Gene	Forward	Backward
Human Nrf2	5'-TGATTCTGACTCCGGCATTT-3'	5'-GCCAAGTAGTGTGTCTCCATAG-3'
Human HO-1	5'-ACCAAGTTCAAGCAGCTCTAC-3'	5'-GCAGTCTTGGCCTCTTCTATC-3'
Human GSTM1	5'-CTTTCCCAATCTGCCCTACTT-3'	5'-CCACACGAATCTTCTCCTCTTC-3'
Human p62	5'-ATTGAGTCCCTCTCCCAGAT-3'	5'-CGCTCCGATGTCAT AGTTCTT-3'
Human NDP52	5'-GCCCATTGACCTAAACAACAAA-3'	5'-CACACCATCCTCATCCACATAG-3'
Human Keap1	5'-TGTCCTCAATCGTCTCCTTTATG-3'	5'-CTCGTTCCTCTCTGGGTAGTA-3'
Human DCAF11 (V1)	5'-CTGTCTTCTCCATTGCTGTCTC-3'	5'-CGGTTCTGTTCTCGGTCAAA-3'
Human DCAF11 (V2)	5'-CCCAGGTACTGGCCTATCT-3'	5'-CATCCCAAGCTCTGTCATTCT-3'
Human β-TrCP	5'-TGGCTCATCTGACAACACTATC-3'	5'-CGAATACAACGCACCAATTCC-3'
Human Hrd1	5'-GACCGTGTGGACTTTATGGAA-3'	5'-GATGCCCAGGAGGAACATAAG-3'
Human ATP6V1H	5'-CCCTGAAGAGAAGCAAGAGATG-3'	5'-TGCAGCATATCATCCACCATAG-3'
Human ATG9b	5'-TCCTTCGATGCGTGGATTAC-3'	5'-GCATCTGACAGGGTCACTTT-3'
Human Lamp1	5'-CGTCAGCAGCAATGTTTATGG-3'	5'-CATGTTCTTAGGGCCACTCTT-3'
Human Cathepsin D	5'-AGTGCTTCACAGTCGTCTTC-3'	5'-GGACTTGTCGCTGTTGTACT-3'
Human TFEB	5'-GGCAACAGTGCTCCCAATA-3'	5'-GACATCGTCCAGACGCATAAT-3'
Human COX-2	5'-TACTGGAAGCCAAGCACTTT-3'	5'-GGACAGCCCTTCACGTTATT-3'
Human GAPDH	5'-GGTGTGAACCATGAGAAGTATGA-3'	5'-GAGTCCTTCCACGATACCAAAG-3'
